# Transcriptomic neuron types vary topographically in function and morphology

**DOI:** 10.1038/s41586-024-08518-2

**Published:** 2025-02-12

**Authors:** Inbal Shainer, Johannes M. Kappel, Eva Laurell, Joseph C. Donovan, Martin W. Schneider, Enrico Kuehn, Irene Arnold-Ammer, Manuel Stemmer, Johannes Larsch, Herwig Baier

**Affiliations:** 1 Max Planck Institute for Biological Intelligence, Martinsried, Germany; 2 Present Address: Faculty of Biology, Technion–Israel Institute of Technology, Haifa, Israel; 3 Present Address: Friedrich Miescher Institute for Biomedical Research, Basel, Switzerland; 4 Present Address: Center for Integrative Genomics, Faculty of Biology and Medicine, University of Lausanne, Lausanne, Switzerland

**Keywords:** Neural circuits, Sensorimotor processing

## Abstract

Neuronal phenotypic traits such as morphology, connectivity and function are dictated, to a large extent, by a specific combination of differentially expressed genes. Clusters of neurons in transcriptomic space correspond to distinct cell types and in some cases—for example, *Caenorhabditis elegans* neurons^1^ and retinal ganglion cells^2–4^—have been shown to share morphology and function. The zebrafish optic tectum is composed of a spatial array of neurons that transforms visual inputs into motor outputs. Although the visuotopic map is continuous, subregions of the tectum are functionally specialized^5,6^. Here, to uncover the cell-type architecture of the tectum, we transcriptionally profiled its neurons, revealing more than 60 cell types that are organized in distinct anatomical layers. We measured the visual responses of thousands of tectal neurons by two-photon calcium imaging and matched them with their transcriptional profiles. Furthermore, we characterized the morphologies of transcriptionally identified neurons using specific transgenic lines. Notably, we found that neurons that are transcriptionally similar can diverge in shape, connectivity and visual responses. Incorporating the spatial coordinates of neurons within the tectal volume revealed functionally and morphologically defined anatomical subclusters within individual transcriptomic clusters. Our findings demonstrate that extrinsic, position-dependent factors expand the phenotypic repertoire of genetically similar neurons.

## Abstract (web summary)

Transcriptomic profiling of neurons from the zebrafish optic tectum identified more than 60 cell types; functional imaging revealed that response tuning, morphology and connectivity of specific cell types vary systematically with the neurons’ positions in the tissue.

## Main

Neurons can be grouped into classes, types, and subtypes according to the combination of genes that they express^7–9^. The cell-type-specific transcriptome (t-type) is believed to encode the genetic instructions for a neuron’s differentiation trajectory during development and thus its morphology (m-type), connectivity and function (f-type). For example, each of the 118 anatomically distinct neuron types in the roundworm *C. elegans* expresses a unique, sparse combination of transcription factors, which regulate downstream genes, thus shaping the neuron’s phenotype and contribution to network function^1,10^. In *Drosophila*, distinct sets of differentially expressed genes are associated with specific axonal or dendritic wiring^11^. Similarly, in the mouse visual cortex, morpho-electric properties measured in tissue slices were found to be relatively homogeneous within individual t-types^12,13^. However, the dogmatic view whereby t-type, m-type and f-type are equivalent is problematic, as functional responses and dendritic or axonal arbor elaborations are often shaped by modulatory influences and plasticity mechanisms^14,15^. For instance, GABAergic (γ-aminobutyric acid-expressing) interneurons in the visual cortex were found to exhibit tuning properties that were dependent on behavioural state rather than t-type^16^. Moreover, mouse cortical neurons of the same t-type may vary strongly in their functional tuning^17^, as well as in their m-type, exhibiting divergent long range projections and local connectivity^18^. It is therefore essential to determine the extrinsic factors that influence the expression of a neuron’s phenotype and how these interact with the transcriptome.

Here we provide evidence that the interplay of transcriptome, development and topography shapes neuronal phenotypes. The zebrafish optic tectum (OT) receives topographically organized input from retinal ganglion cells (RGCs) along the anterior–posterior and dorsal–ventral axes^19,20^. In its superficial-to-deep (SD) dimension, orthogonal to the retinotopic axes, the OT contains a neuropil layer in which RGC axons form synapses with the dendrites of tectal neurons, and a cell body layer, the stratum periventriculare (SPV). Cellular birthdating studies have shown that newborn neurons are initially added radially to the OT from the periventricular zone and later to the posterior margins^21,22^, with older neurons being gradually displaced into the SPV as they mature and extend neurites into the neuropil^23,24^. Extrinsic factors, including morphogens, chemotropic and cell-surface factors, vary in concentration and availability over the course of development and along all three axes of the OT^25^. We provide evidence here that such cues might affect the phenotype of OT neurons in a location-dependent manner.

## Tectal cell types are molecularly diverse

**Fig. 1:**
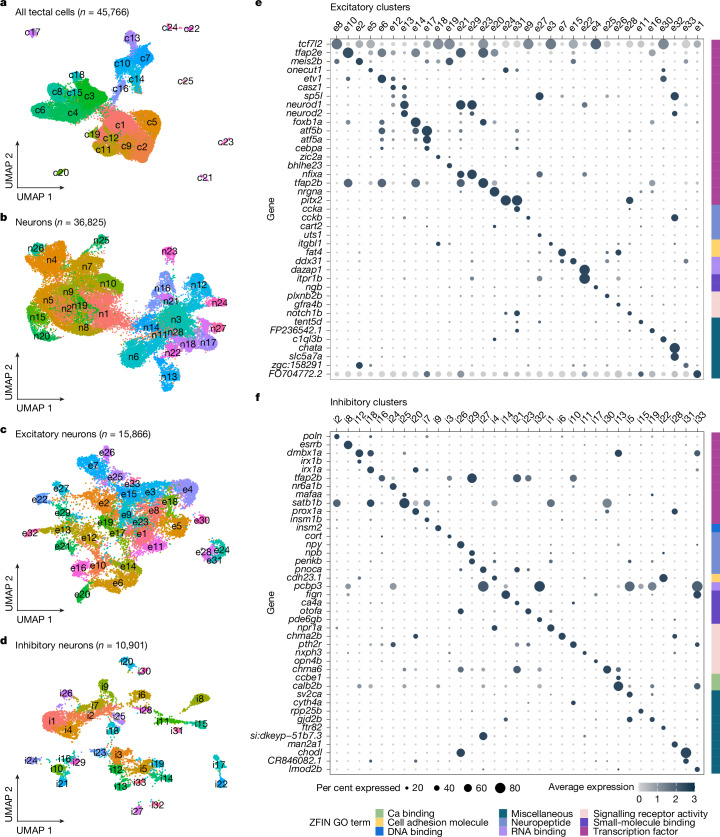
scRNA-seq of the OT reveals a multitude of neuronal types. **a**, Sequenced tectal cells clustered according to similarity of gene expression; 25 distinct clusters were identified. Each dot represents a single cell, colour coded according to cluster identity. **b**, Postmitotic neurons (clusters expressing *elavl3*) were subsetted and reclustered to further identify the various types of excitatory and inhibitory neurons. **c**, Excitatory neurons reclustered according to similarity of gene expression. **d**, Inhibitory neurons reclustered according to similarity of gene expression. **e**, Dot plot of the highly differentially expressed genes for each of the excitatory cell types (clusters e1–e33). Differentially expressed genes were grouped according to their molecular function and annotated according to ZFIN Gene Ontology terminology^58^. **f**, Dot plot of the highly differentially expressed genes for each of the inhibitory cell types (clusters: i1–i33).

**Extended Data Fig. 1:**
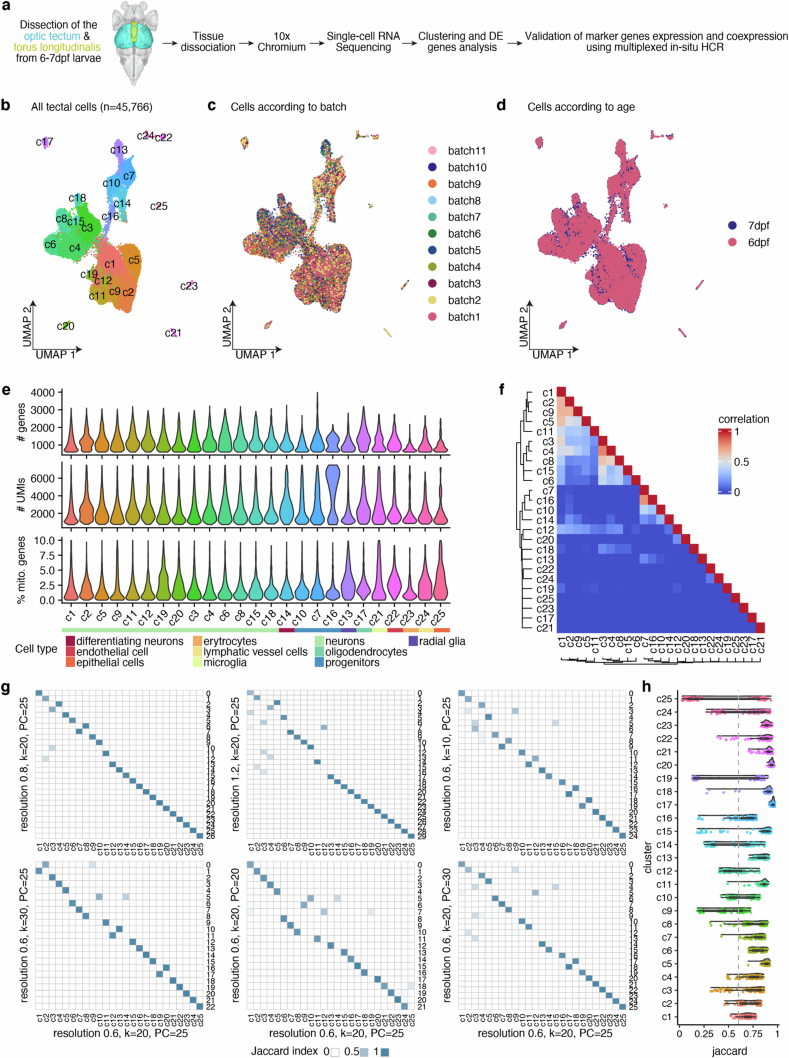
QC of the scRNA-Seq clustering analysis. **a**, Experimental procedure: optic tectum and torus longitudinalis (TL) were dissected from 6-7 dpf WT larvae in eleven experimental batches. The cells were dissociated and single-cell barcoded cDNA was generated using the 10x Chromium system and sequenced. Cells were clustered into t-types according to the similarity of gene expression. **b**, After batch correction using Harmony^63^ and removal of immediate early genes (see Methods), we identified a total of 25 clusters. Each dot represents a single cell, colour coded according to the cluster. **c**, Same UMAP as in **b**, colour coded according to the batch. Clusters include cells from all batches. **d**, Same UMAP as in **b**, colour coded according to the larvae age. Clusters include cells from both 6 and 7 dpf larvae. **e**, QC metric of the sequenced cells after filtration, according to cluster. Cells were filtered (see Methods) in order to remove outliers that contain either low or high number of genes and UMI (representing either poorly sequenced cells or doublets), as well as cells containing high percentage of mitochondrial genes (representing stressed cells). Different cell types contained different levels of genes and UMIs, matching their biological profile (for example high levels of UMIs in some of the progenitor cells). **f**, The similarity between clusters was assessed by correlating the specificity scores of the top markers associated with each cluster (see Methods). The vast majority of clusters showed low correlation with other clusters, representing unique t-types. **g**, The robustness of the clusters was assessed by measuring the overlap of cells grouped together under different clustering parameters in comparison to the parameters used (see Methods). **h**, Jaccard raincloud plot showing cluster stability. 80% of the data were subsampled 100 times, with pairwise Jaccard indices calculated for each cluster before and after subsampling and reclustering (see Methods). In most instances, we observed that clusters with small numbers of cells were scored low, as a result of infrequent sampling of those cells. Box plots include all data points. The lower and upper hinges represent the first and third quartiles, respectively. Whiskers extend to the largest and smallest values within 1.5 × IQR from each hinge.

**Extended Data Fig. 2:**
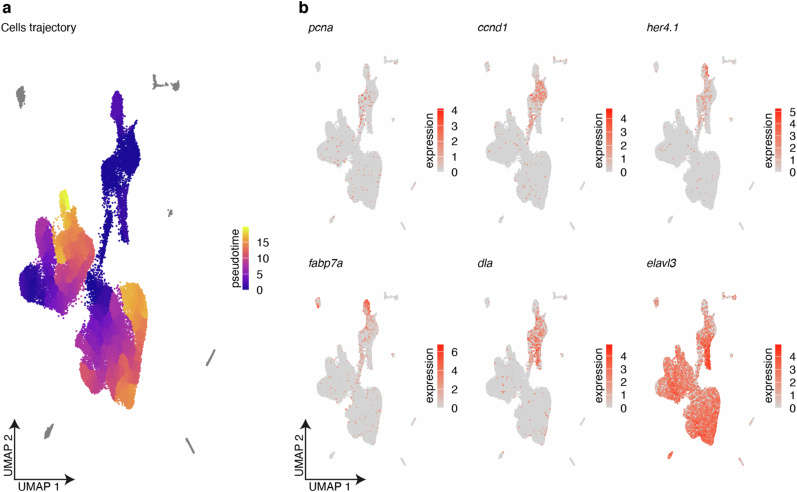
Pseudotime analysis. **a**, Pseudotime analysis was performed with Monocle3^86^. Cells expressing proliferating cell nuclear antigen (*pcna*), a key component of the DNA replication machinery and a marker of the G1 phase^87^, and belonging to clusters c7, c10, and c16, were identified as progenitor cells and defined as the root point. Cells are colour coded according to their pseudotemporal ordering. **b**, Expression patterns of selected marker genes, representing the different stages of proliferation and differentiation. The marker *ccnd1* is a member of the cyclin family that regulates the cell-cycle transition from G1 to S phase^88^. The markers *fabp7a* and *her4.1* are typically expressed in radial glia^89,90^. Delta A (*dla*) regulates cell fate specification^91^, and *elavl3* is expressed in postmitotic neurons.

**Extended Data Fig. 3:**
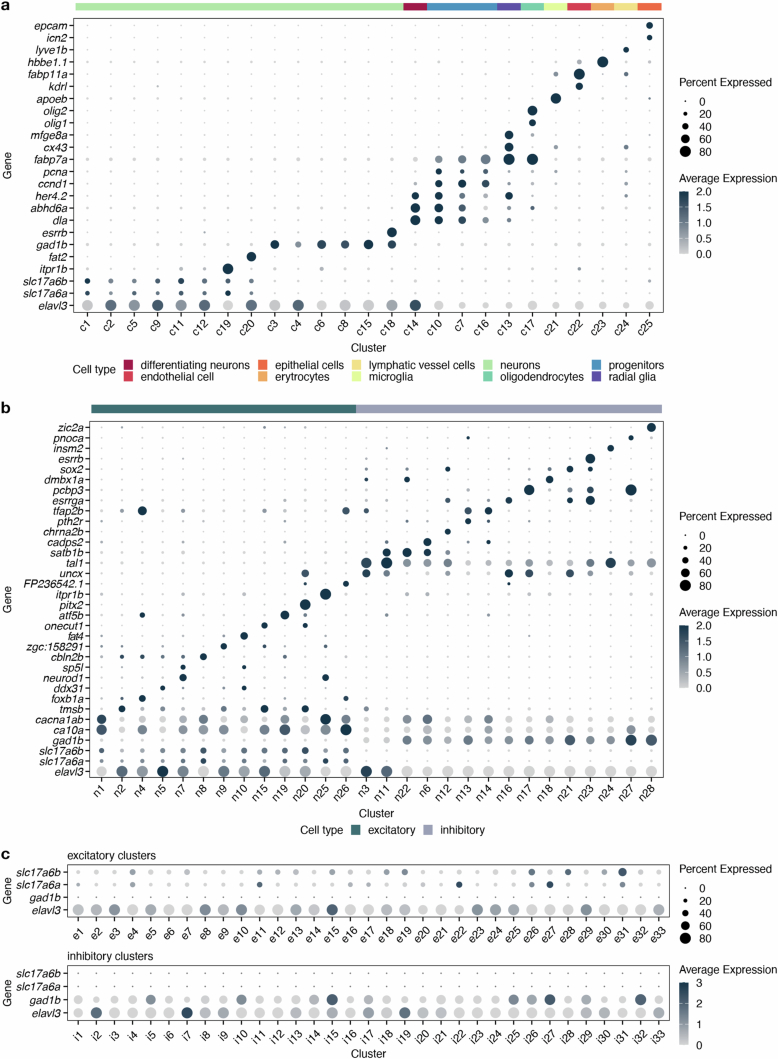
Cell type classification. **a**, Dotplot of the highly DE genes for each tectal cluster. Clusters were grouped according to cell function. Cluster c20 cells expressed torus longitudinalis marker genes and were therefore omitted from downstream tectum analyses. **b**, Dotplot of the highly DE genes for each neuronal cluster. Clusters were grouped according to transmitter use. Cluster n3 highly expresses the transcription factors *uncx* and *tal1*, which are typically expressed in differentiating neurons^92–94^. **c**, Based on *gad1b*, *slc17a6a* (*vglut2b*) and *slc17a6b* (*vglut2a*) expression, we separated the inhibitory and excitatory clusters and reclustered them (see Methods).

To characterize the t-type composition of the zebrafish OT, we performed droplet-based single-cell RNA sequencing (scRNA-seq; Extended Data Fig. 1 and Methods) of the OT of larvae at 6–7 days post fertilization (dpf). We sequenced 45,766 cells, which corresponds to more than 7× coverage of cells in a single tectal hemisphere^5^ (approximately 6,000 neurons), and grouped them according to their transcriptomes into 25 major clusters (Fig. 1a and Extended Data Fig. 1). On the basis of differentially expressed, cluster-specific genes (Extended Data Figs. 2 and 3 and Supplementary Table 1), we identified 3 clusters of progenitor cells, 1 cluster of radial glia, 14 clusters of neurons and 6 additional non-neuronal populations (Extended Data Figs. 2 and 3).

**Fig. 2:**
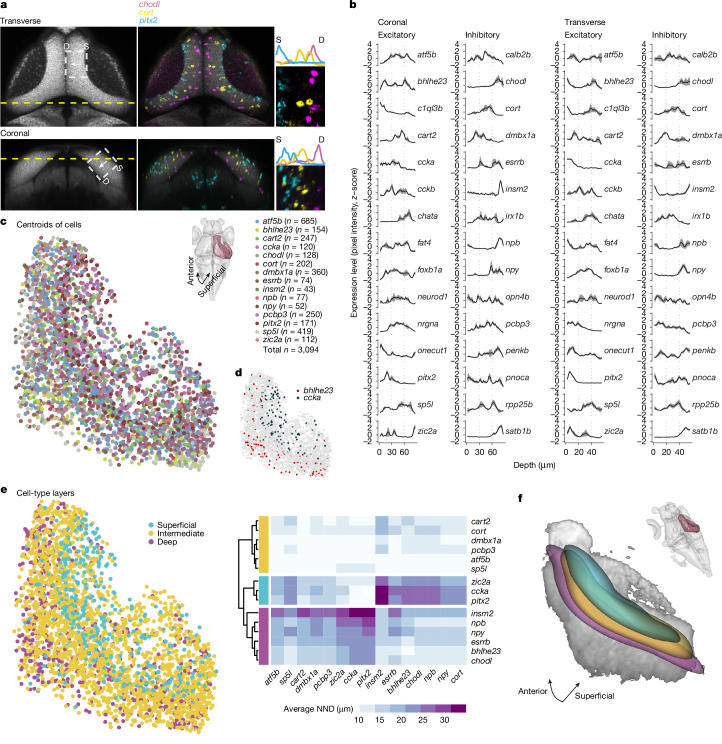
Cell bodies of transcriptomic clusters form anatomical layers in the SPV orthogonal to the retinotopic map. **a**, Multiplexed in situ HCR of selected marker genes were registered to the standard brain atlas at mapzebrain.org. We measured expression level by examining the pixel intensity profile of each gene in the same transverse (top, projection of planes z289–z291) and coronal (left, projection of planes x463–x465) section. Left, the area in which pixel intensity is measured is indicated with a white dashed rectangle and confined to the SPV (S, superficial; D, deep). Middle and right, expression pattern example in a single fish. **b**, The *z*-scored pixel intensity of selected inhibitory and excitatory marker genes. The black line represents the average (*n* = 3 fish, simple moving average with window size = 3); the grey shaded region represents the s.e.m. **c**, Bottom left, centroids of cells expressing selected t-type markers located within the SPV. Top right, dorsal view with the right hemisphere SPV highlighted in burgundy, representing the labelled area of the centroids in the tectal coordinate system. **d**, The labelled centroids of cells expressing *bhlhe23* and *ccka*, demonstrating the segregation of these cells along the tectum in 3D. **e**, The distance from each cell of a given t-type to its nearest neighbour (NND) of all other t-types was measured in 3D. Hierarchical clustering of the average NND for each t-type revealed three molecular layers in the SPV. **f**, Bottom left, 3D visualization of thresholded gaussian kernel densities for the three molecular layers from **e**. Top right, the viewing angle relative to the whole brain.

**Extended Data Fig. 4:**
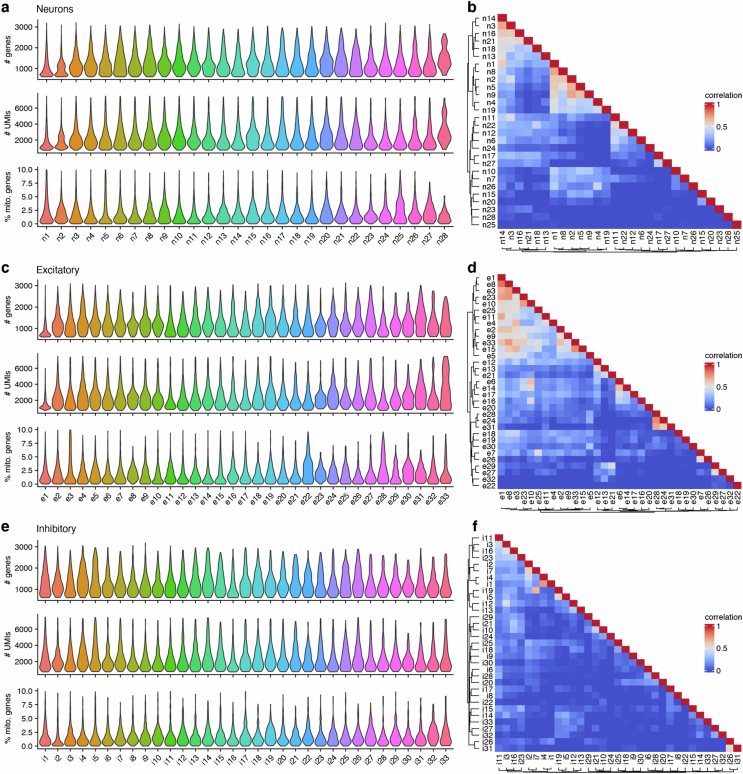
QC metrics for clustering of excitatory and inhibitory cells. **a**, Distribution of gene count, UMI count and percentage of mitochondrial genes per neuronal cluster. **b**, The similarity between neuronal clusters was assessed by correlating the specificity scores of the top markers associated with that cluster (see Methods). **c**, Same as **a**, per excitatory cluster. **d**, Same as **b**, for the excitatory clusters. **e**, Same as **a** per inhibitory cluster. **f**, Same as **b**, for the inhibitory clusters.

**Extended Data Fig. 5:**
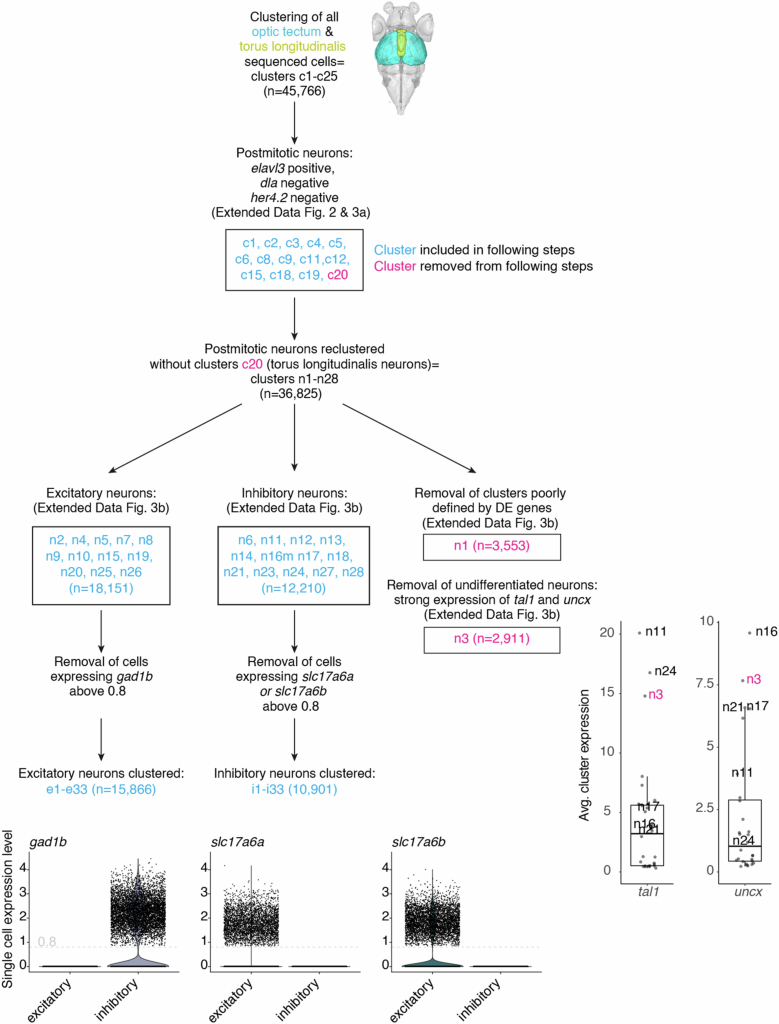
Summary of scRNA-seq clustering steps. Average cluster expression box plot includes all cluster points. The lower and upper hinges represent the first and third quartiles, respectively. Whiskers extend to the largest and smallest values within 1.5 × IQR from each hinge.

The postmitotic neurons (36,825 cells expressing *elavl3*) were reclustered (Fig. 1b and Extended Data Figs. 2–4). Poorly annotated clusters were removed (Extended Data Fig. 5), and the remaining clusters were separated into excitatory (*gad1b*^−^, *slc17a6a*^+^ and *slc17a6b*^+^) and inhibitory (*gad1b*^+^, *slc17a6a*^−^ and *slc17a6b*^−^) neurons (Fig. 2c,d and Extended Data Figs. 3c and 4). This resulted in 33 excitatory t-types (clusters: e1–e33; total number of cells = 15,866) and 33 inhibitory t-types (clusters: i1–i33; total number of cells = 10,901). For 20 neuronal types, we identified individual differentially expressed genes that serve as mutually exclusive markers; for the remaining types, sparse combinations of differentially expressed genes sufficed for an unambiguous definition (Fig. 1e,f and Supplementary Table 1). Overall, we identified 66 neuronal t-types in the larval zebrafish OT (Extended Data Fig. 5). The composition of cell types and marker genes that we identified differed from a recent study that characterized the zebrafish OT^26^. In that study, fluorescence-activated cell sorting of tectal neurons from a specific enhancer trap line was used before the scRNA-seq, which biased the data towards the fraction of cells labelled by that line^26^. This most probably resulted in an incomplete coverage of the OT cell types and the observed higher proportion of immature neuronal populations. Our approach was designed to encompass all tectal cells, which allowed us to identify a richer repertoire of neuronal and non-neuronal populations.

## t-types are arrayed topographically

**Extended Data Fig. 6:**
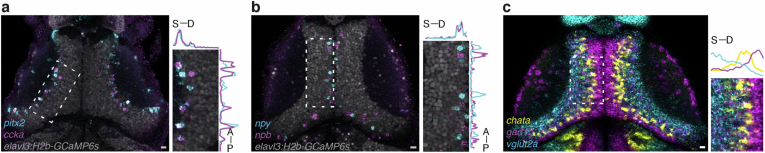
Multiplexed in situ HCR labelling of cell type markers. We applied in situ HCR to analyse and validate the spatial arrangement of the cell types’ marker genes. **a**, The marker gene *ccka* was co-expressed with *pitx2* in cluster e31 but not in cluster e24. An example of a single animal. Dashed square area is enlarged and the pixel intensity (z-scored) is plotted, demonstrating both the co-expression and the localization to the superficial part of the SPV (S; superficial, D; deep). Scale bar = 10 µm. **b**, The expression of the neuropeptides *npb*, marker of clusters i22 and i29, and *npy*, marker of cluster i26 was mutually exclusive. In situ HCR analysis demonstrated the localization to the deep sublayer of the SPV. An example of a single animal. Scale bar = 10 µm. **c**, Registered averaged in situ data of *gad1b*, *vglut2a* (*slc17a6b*), and *chata* (n = 3 for each gene) revealed that glutamatergic, cholinergic, and GABAergic neurons are segregated along the tectal superficial to deep axis. Scale bar = 10 µm.

**Extended Data Fig. 7:**
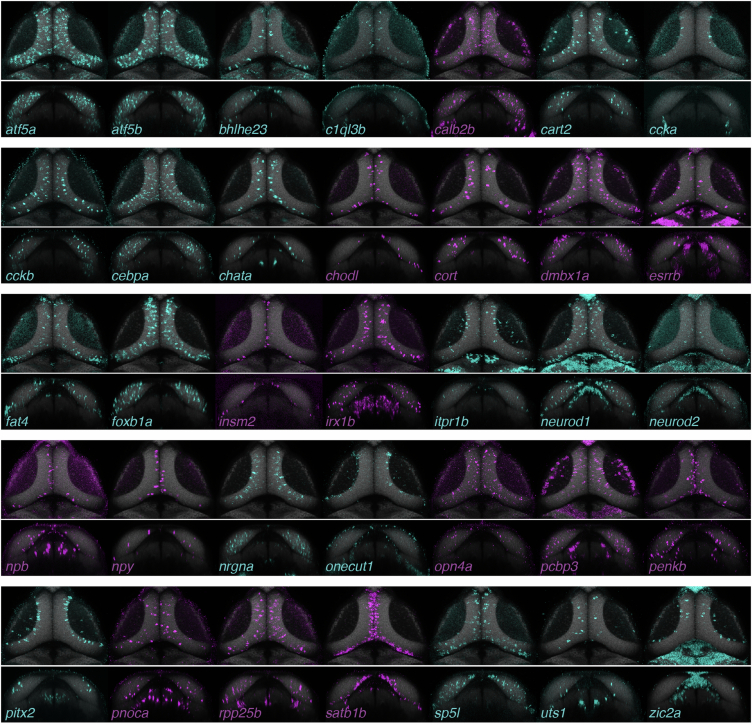
Expression patterns of marker genes. Registered in situ HCR data for the examined cell type markers. Projection of transverse planes z289-z291 and coronal planes x463-x465 of a single animal for each gene. Excitatory markers are shown in cyan; inhibitory markers are shown in magenta. All the data can be viewed and downloaded at mapzebrain.org^28^.

To test whether t-types are spatially organized relative to the three axes of the OT volume, we selected differentially expressed genes that were expressed in a single or a small number of clusters (Fig. 1) and examined their spatial expression patterns using multiplexed RNA in situ hybridization chain reaction (HCR). We coregistered the HCR patterns within the standard coordinates of the mapzebrain atlas^27,28^ (https://mapzebrain.org) and measured their expression levels at the same transverse and coronal sections (Fig. 2 and Extended Data Figs. 6 and 7).

We found that genetically identified neurons are enriched in specific domains along the SD axis. Most prominently, GABAergic and glutamatergic neurons largely populate the deepest and most superficial layer of the SPV, respectively, with cholinergic neurons sandwiched between them (Extended Data Fig. 6). Individual excitatory and inhibitory t-types generally follow this rule, but can also break it. For example, the excitatory marker *bhlhe23* is expressed in deep SPV neurons, where it is surrounded by inhibitory *insm2*, *chodl*, *npb* and *npy* neurons (Fig. 2 and Extended Data Figs. 6 and 7). The excitatory markers *ccka*, *onecut1*, *pitx2* and *zic2*a are restricted to superficially located neurons in the SPV (Fig. 2 and Extended Data Figs. 6 and 7), whereas *cort*, *cckb*, and *irx1b* are expressed by neurons in the middle of the SPV (Fig. 2 and Extended Data Fig. 7). Some t-types are present in all SPV regions, namely those expressing *atf5b* and *sp5l*, whereas the inhibitory markers *esrrb* and *rpp25b* show a two-layer expression pattern (Fig. 2). We rarely found neurons expressing the same marker directly adjacent to each other (Fig. 2 and Extended Data Figs. 5 and 6), suggesting that neurons of the same type form a mosaic in the SPV, similar to individual RGC types in the retina.

**Extended Data Fig. 8:**
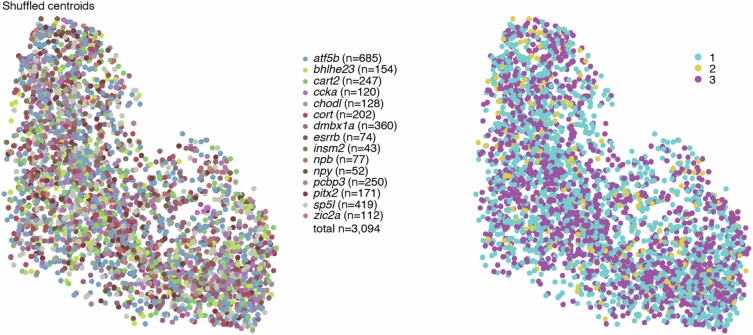
Spatial organization of shuffled labels. Shuffling the centroid labels of tectum cell types results in the loss of the observed molecular layers within the tectum.

To obtain a holistic picture of OT cell-type architecture, we manually labelled the neurons’ centroid positions (Fig. 2c,d), and measured the nearest neighbour distances (NNDs) in 3D, between each centroid in a given t-type to its nearest neighbours of all other t-types. We then performed hierarchical clustering on the mean NND vector for each t-type (Fig. 2e). The spatial organization of the clustered NND vectors divided the SPV into three distinct layers: superficial, intermediate and deep (Fig. 2e,f), illustrating locally neighbouring t-types. This organization disappeared with shuffled labels (Extended Data Fig. 8). Given that the superficial cells of the SPV are born before the deep cells^22^, this finding suggests that similar to the retina^29^, cell classes and cell types develop in a predetermined order, with excitatory t-types generally preceding inhibitory t-types (Fig. 2 and Extended Data Fig. 6c).

## Combinatorial patterns refine t-types by axes

**Fig. 3:**
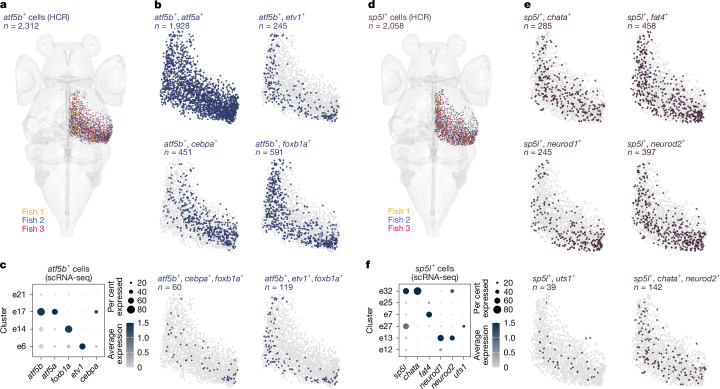
Combinatorial expression of t-type marker genes separates subtypes along the anatomical axes of the OT. **a**, *atf5b*-expressing cells were labelled in three individual larvae and patterns were coregistered (total *n* = 2,312 cells). **b**, Combinatorial expression of marker genes within the *atf5b*-expressing cells. *atf5b*^*+*^ *etv1*^+^ cells were found in the deep SPV region, whereas *atf5b*^*+*^ *cebpa*^+^ cells were localized to the superficial region with highest density towards the posterior zone. **c**, scRNA-seq data for clusters expressing *atf5b*. **d**, *sp5l*-expressing cells were labelled from 3 individual larvae (total *n* = 2,058). **e**, Combinatorial expression of marker genes among the *sp5l*-expressing cells. *sp5l*^*+*^ *chata*^+^ cells were found in the intermediate SPV region. *sp5l*^+^ *neurod1*^+^ cells were found in the intermediate and deep regions, with highest density towards the posterior zone. *sp5l*^*+*^* uts1*^+^ cells represented a rare population located in the intermediate SPV region. **f**, scRNA-seq data for clusters expressing *sp5l*.

The spatially analysed differentially expressed genes included markers that are expressed in a single cluster or a small number of clusters. To further examine the spatial organization of transcriptomic subtypes defined by a combination of marker genes, we performed iterative multiplexed HCR labelling of submarkers for cells expressing *atf5b* and *sp5l*, respectively, and examined their spatial positioning. We focused on a combination of transcription factors and other unique effector genes (encoding mainly neuropeptides and proteins for neurotransmitter synthesis or transport^30^). The *atf5b* gene was strongly expressed in cluster e17 and weakly detected in several clusters, including e6, e14 and e21 (Figs. 1 and 3). On the basis of cluster e17 markers, we explored the anatomical arrangement of *atf5b* t-types. Neurons that co-expressed *atf5b* and the transcription factor *foxb1a* or *etv1* were separated along the SD axis, similar to the molecular layers, whereas co-expression with *cebpa*, which encodes an inhibitor of cell proliferation^31^, had higher density in the superficial posterior zone (Fig. 3a,b). Similarly, neurons that co-expressed *sp5l* with the effector genes *uts1* and *chata* were separated within the intermediate molecular layer, whereas co-expression with the neurogenic factor *neurod1*^32,33^ was concentrated in the posterior zone (Fig. 3d,e). Overall, we find that clusters defined by either a single marker or a combinatorial code were spatially segregated along the SD axis, whereas distribution along the SPV anterior–posterior axis was related to neurogenic factors and the progression of neuronal differentiation from the posterior marginal proliferative zone^23,24^.

## Visual responses diverge within individual t-types

**Fig. 4:**
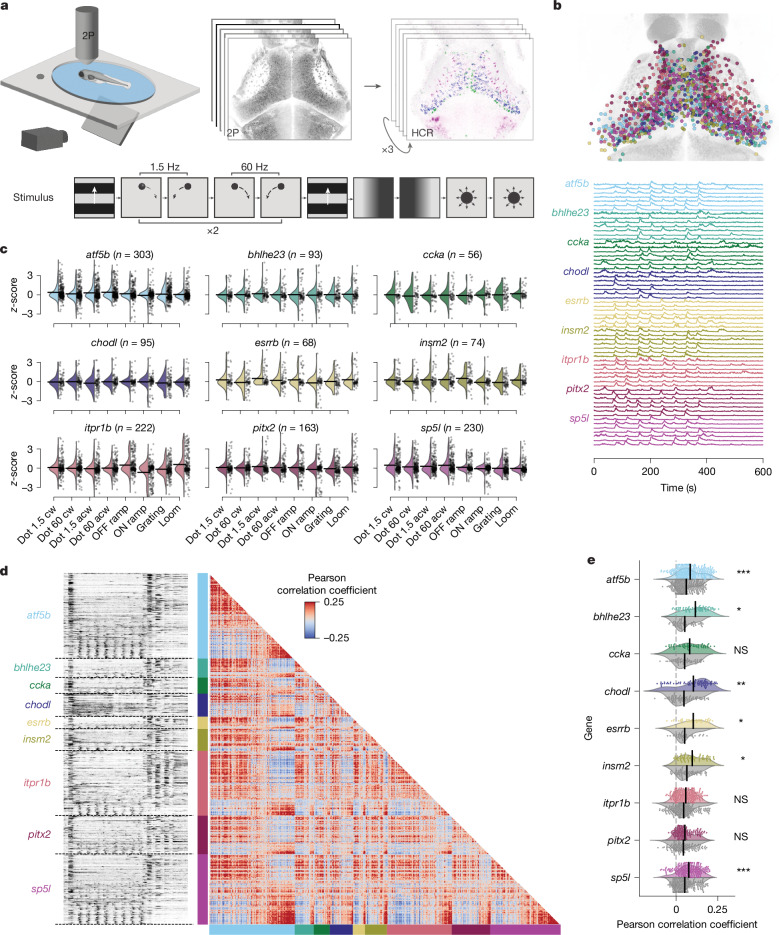
Neurons of a specific t-type show diverse visual responses and form coherent functional subclusters. **a**, Experimental procedure: 6 dpf larvae were exposed to a battery of visual stimuli while volumetric functional 2-photon calcium imaging (2P) was performed covering most neurons of the tectum. The larvae were then stained in consecutive rounds for up to six marker gene mRNAs using HCR labelling. The larva illustration was modified from ref. ^59^. **b**, Top, result of aligning functional and HCR brain volumes and registering all ROIs that overlap with one of 9 marker genes into a common anatomical reference frame (1,304 ROIs, *n* = 6). Bottom, example traces of each labelled t-type selected for responsiveness to local motion. **c**, Average t-type response scores to the visual stimulus sequence scaled to unit variance and zero mean of overall tectal population. Most t-types showed increased or decreased responses to at least two different stimuli. acw, anticlockwise; cw, clockwise. **d**, Left, raw calcium traces of all 1,304 t-type+ functional ROIs sorted by first t-type and within t-type by response score to local motion. Responses to global motion (beginning, end) and local motion (middle section) are visible in all t-types. Right, correlation matrix of all pairwise correlations using Pearson correlation coefficient. Red and blue clusters of positively and negatively correlated neurons are found between and within all t-types. **e**, Mean pairwise correlations of each neuron with all neurons of the same t-type (colour) and all neurons (grey) of other t-types. Within t-type correlation is significantly increased for six out of nine tested t-types. Two-sided Student’s *t*-test, Bonferroni-corrected. *P* values: *atf5b*, 3.053 × 10^−4^; *bhlhe23*, 1.367 × 10^−2^; *ccka*, 1.443 × 10^−1^; *chodl*, 5.043 × 10^−3^; *esrrb*, 3.769 × 10^−2^; *insm2*, 1.556 × 10^−2^; *itpr1b*, 1.653 × 10^−1^; *pitx2,* 3.703 × 10^0^ *sp5l*: 1.076 × 10^−4^. *P < 0.05, **P < 0.01, ***P < 0.001. NS, not significant.

**Extended Data Fig. 9:**
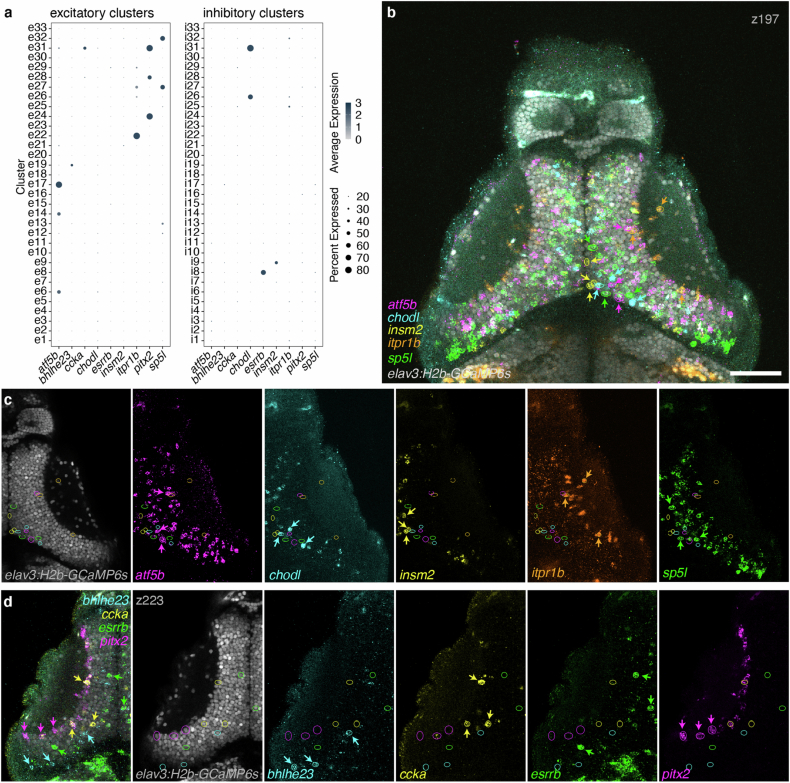
HCR labelling of marker genes post calcium imaging. **a**, HCR was performed on a set of nine marker genes. The expression levels of these genes measured by scRNA-Seq showing their expression is restricted to one or a few non-overlapping clusters, except for *ccka* that overlaps with *pitx2* expression. **b**, Multiplexed iterative HCR labelling post functional imaging. A single fish example out of 3 studied with a similar gene set labelled. Five genes were labelled and registered. Similar to the scRNA-Seq data, these genes were not co-expressed in the same cells, representing different tectal t-type populations. Circles and arrowheads point to example cells shown in **C**, colour coded similarly to the gene HCR colour. Single z-plane is shown; scale bar = 50 µm. **c**, Same view as **b**, splitted according to the genes labelled. **d**, Example of another multiplexed iterative HCR post functional imaging of a single animal out of 3 studied, with a different set of labelled cell type marker genes.

We next examined whether neurons of a given t-type shared selectivity to one or multiple sensory or motor features. To this end, we recorded calcium activity by 2-photon volumetric imaging in tectal neurons of *elavl3:H2B-GCaMP6s* transgenic larvae that were exposed to a sequence of behaviourally relevant visual stimuli^5,34^. The stimuli included both local and global motion cues: dots moving in different directions, either continuously or in a saltatory fashion (distinguishing a neutral or prey-like cue from a social cue), a forward moving grating (evoking optomotor responses), a looming black disk (simulating a predator or an object on a collision course) and ambient luminance changes (Fig. 4a). To determine the t-type for each of the recorded neurons, we subsequently performed iterative multiplexed HCR labelling of up to six mutually exclusive marker genes (except for *pitx2* and *ccka*; Extended Data Fig. 9). By co-registering the two volumetric datasets, we could unambiguously assign a specific t-type to 1,304 functionally characterized neurons from 6 fish (Fig. 4b and Methods).

We found that average t-type responses had increased scores for at least two stimuli of the set compared with the population mean response (Fig. 4c). For example, moving dots and moving gratings evoked strong activity in the *atf5b* type, whereas *itpr1b* neurons scored highest for looming and OFF ramp stimuli (Fig. 4c). To test whether individual neurons within the same t-type or between t-types have similar functional responses, we correlated calcium traces of all t-type+ neurons with each other across animals (Fig. 4d). A correlation matrix of raw calcium traces, sorted by t-type, and within t-type by overall response score to local motion stimuli, revealed stereotyped clusters of positive and negative pairwise correlations within and between all t-types (Fig. 4d). This indicates a functional diversity within each t-type, varying mostly in the relative frequency of local-motion- and global-motion-tuned neurons. Neurons co-expressing *pitx2* and *ccka*, although identified in only 12 neurons, showed a mixed functional tuning for local and global motion, similar to neurons characterized by a single marker gene (Extended Data Fig. 11e). Mean pairwise correlations of calcium responses were significantly higher within t-types than between t-types for six out of nine marker genes tested (Fig. 4e). We conclude that neurons of the same t-type are not coherent in their visual tuning but are, on average, functionally more similar when compared to other t-types. In line with our findings, distinct t-types in the superior colliculus, the mammalian homologue of the OT, also showed heterogeneous specificity in their visual responses^35^.

## Transcriptome and position govern function

**Fig. 5:**
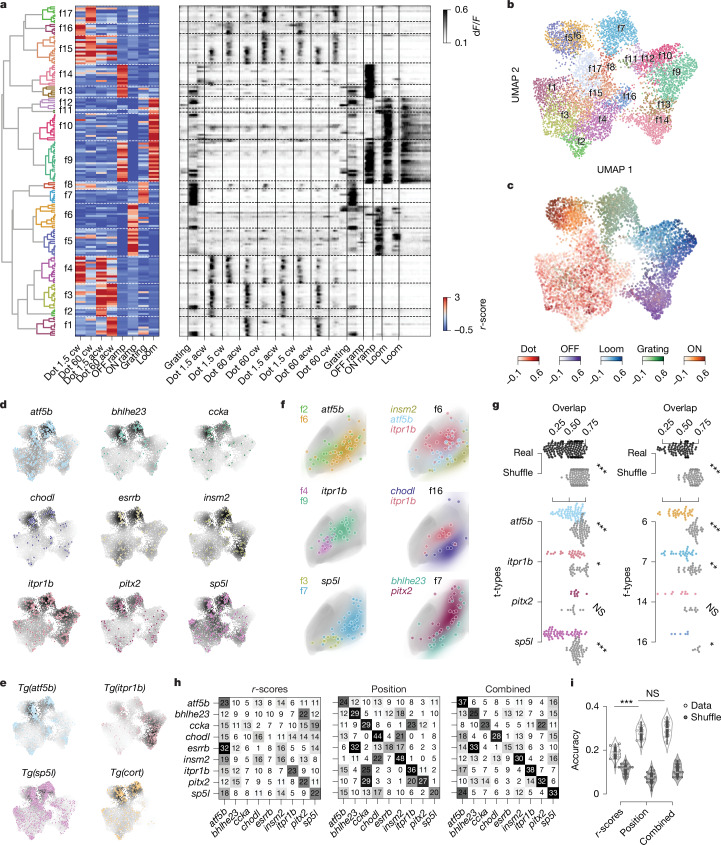
Localization in functional and anatomical space varies between t-types. **a**, Hierarchical clustering of all strongly responding tectal neurons (*n* = 7,094) to identify functional cell types (f-types). Left, dendrogram of clustering all 169 exemplars that were identified using affinity propagation. Middle, heat map showing response vectors of all exemplars based on visual stimuli. Functional clusters and exemplars are primarily divided into local (clusters 1–4 and 15–17) versus global (clusters 5–14) motion responses. Right, raw calcium traces of all exemplars. **b**, UMAP embedding based on response vectors, overlaid with colour code according to f-type assignment. **c**, UMAP embedding of all responding neurons from **b**, with five different colour codes based on the preferred respective responses of the five superclusters. **d**, t-types accumulate locally in functional space. Response vectors of each transcriptomically identified neuron transformed into the UMAP embedding. The colour code of UMAP represents the kernel density estimate of the respective t-type. **e**, Response vectors of neurons recorded in transgenic fish, mapped into the same UMAP embedding largely confirm the enrichments observed in **d**. **f**, Anatomical localization of t/f-clusters within t-types and f-types. Dots represent individual ROIs, coloured areas show KDEs of t/f-clusters. ROIs of the same t-type (left) and f-type (right) comprise anatomically separated clusters based on functional (left) and transcriptomic (right) identity. All ROIs mirrored to the left tectal hemisphere. t-type is based on HCR labelling. **g**, t/f-clusters are significantly separated in anatomical tectal space within t-types and f-types. Left, pairwise KDE overlap values for cell types of different functional clusters for real data and shuffled t-type labels. Right, as left but for different t-types within functional clusters. Mean pairwise overlap values of t/f-clusters across t-types and across f-types are significantly lower than respective shuffled controls, respectively, indicating anatomical separation of cell types within functional clusters. Two-sided Mann–Whitney *U*-test, Bonferroni-corrected. *P* values: t-types overall, 3.353 × 10^−22^; *atf5b*, 5.598 × 10^−11^; *itpr1b*, 2.203 × 10^−4^; *pitx2*, 9.370 × 10^−1^; *sp5l*, 2.662 × 10^−9^; clusters overall, 2.230 × 10^−14^; c6, 1.822 × 10^−7^; c7, 2.641 × 10^−4^; c14, 4.032 × 10^−3^; c16, 3.463 × 10^−2^. **h**, Confusion matrices of three SVM classifiers predicting transcriptomic identity on the basis of functional response vectors (r-scores), cell body position or both combined. Numbers and saturation indicate the true-positive rate. Predictive performance increases from left to right, indicated by the saturation of the diagonal (true-positive predictions per cell type). **i**, Accuracy of SVM classifier performances from **h** (grey dots and violin plots). Classifiers could recover t-type identity based on functional response vectors in about one out of five cases (accuracy = 0.187 ± 0.027, *n* = 19), for anatomical space accuracy was significantly higher (accuracy = 0.276 ± 0.030, *n* = 19, two-sided Mann–Whitney *U*-test; *P* value = 1.358 × 10^−7^). Combining both spaces did not significantly improve performance compared with anatomical space alone (accuracy = 0.296 ± 0.036, *n* = 19, two-sided Mann–Whitney *U*-test, *P* value = 0.113). For all classifiers, negative controls with shuffled cell-type labels resulted in significantly lower performance (grey dots and violin plots; two-sided Mann–Whitney *U*-test, *P* values: r-score, 1.881 × 10^−7^; position, 6.532 × 10^−8^; combined, 6.532 × 10^−8^).

**Extended Data Fig. 10:**
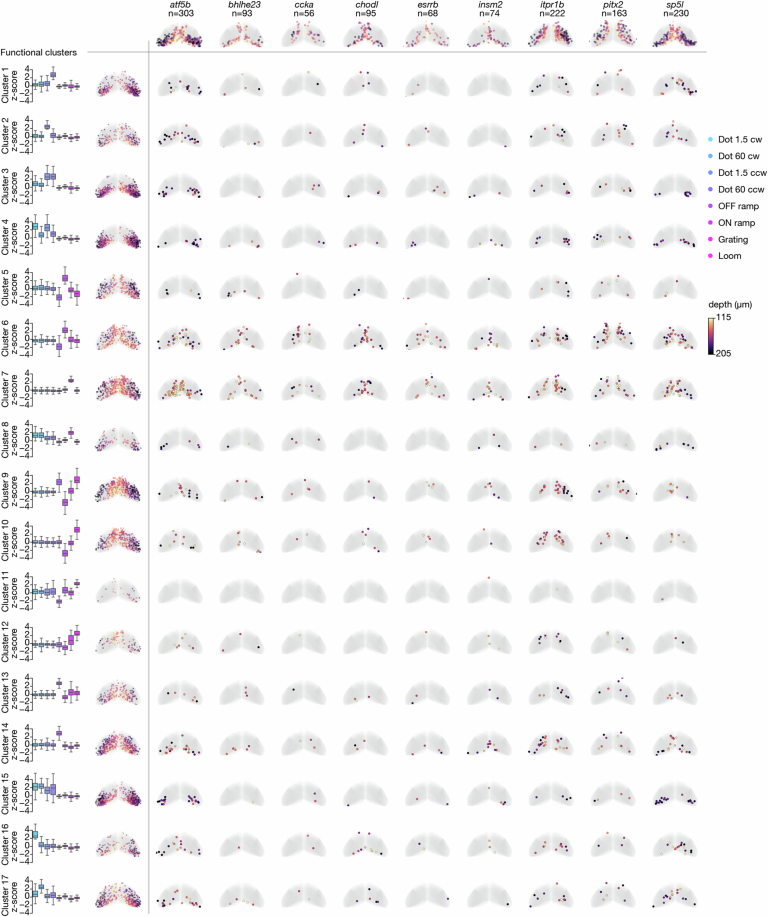
t-types comprise anatomically localized functional clusters. Left: response score distributions of each f-type scaled to tectal population mean (colour coded according to stimulus type) along with their anatomical distribution. Box plots represent the first and third quartiles, respectively. Whiskers extend to the largest and smallest values within 1.5 × IQR from each hinge excluding outliers. Top: anatomical distribution of the t-types recorded (colour coded according to tissue depth). Inner matrix: anatomical localization of HCR labelled and recorded neurons separated into assigned f-types (rows) and t-types (columns). Colour code depicts tissue depth.

**Extended Data Fig. 11:**
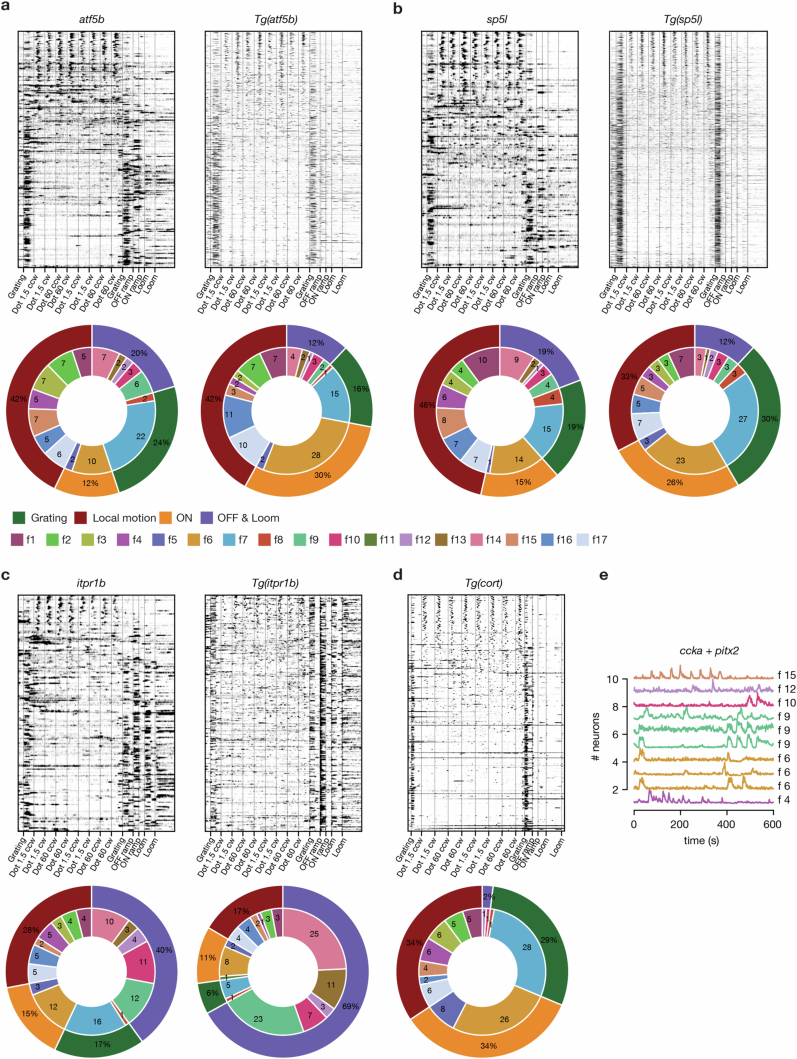
Comparison of functional responses between HCR labelled t-types and the corresponding transgenic lines. **a**, Top: raw calcium traces of HCR labelled (left, n = 303) and transgenic (right, n = 1211) *atf5b+* neurons, sorted by overall response score to local motion in descending order. Bottom: relative fractions of functional types (inner ring, according to Fig. 5a) and groups (outer ring, according to Fig. 5c) within *atf5b*+ neurons for HCR labelled (left) and transgenic (right) cells. **b**, Same as **a**, but for HCR labeled (left, n = 229) and transgenic (right, n = 2250) *sp5l*+ neurons. **c**, Same as **a**, but for HCR labelled (left, n = 222) and transgenic (right, n = 332) *itpr1b*+ neurons. **d**, Same as **a**, but for transgenic *cort*+ neurons (n = 450). **e**, Raw traces and functional types of HCR labeled *ccka*+ *pitx2*+ neurons.

Next, we tested whether any functional response type (f-type) in the OT was linked to one specific t-type. To this end, we first classified functional responses by clustering the highest-responding tectal neurons to each stimulus, excluding HCR-labelled neurons (*n* = 7,094). This resulted in 17 distinct f-types (Fig. 5a). The response vectors formed five superclusters in a uniform manifold approximation and projection (UMAP) embedding, corresponding to broad classes of neurons tuned to local motion, ON, OFF, looming and grating motion, respectively (Fig. 5b,c). We then assigned one of the 17 f-types to each HCR-labelled and functionally profiled region of interest (ROI; Extended Data Fig. 10). The resulting distribution of f-types within t-types revealed that no f-type was specific to or highly overrepresented in one t-type. In parallel, we investigated whether any t-type was overrepresented in a functional cluster or supercluster. (Fig. 5d and Extended Data Fig. 11). Transforming all HCR-labelled functional ROIs into the UMAP embedding revealed that specific t-types were locally enriched within f-type superclusters. A similar result was obtained when mapping response vectors from neurons in transgenic lines expressing GCaMP6s under the control of the *atf5b*, *cort*, *itpr1b* or *sp5l* regulatory regions (Fig. 5e, Extended Data Fig. 11 and Methods), demonstrating the validity of the HCR labelling approach.

We speculated that the observed diversity of discrete f-types within t-types had its origin in the anatomical localization of individual cell bodies. To test this hypothesis, we grouped recorded neurons of the same t-type and same f-type into t/f-clusters and determined their anatomical distributions. The t/f-clusters within t-types were often separated along the anterior–posterior anatomical axis: For instance, f-types 4 and 9 were almost completely non-overlapping in *itpr1b*^+^ neurons (Fig. 5f). To quantify this observation, we computed the gaussian kernel density estimate (KDE) of anatomical cell body distributions for each t/f-cluster and measured the pairwise spatial overlap of t/f-cluster KDEs within t-types and f-types, respectively. We found that t/f-clusters showed larger anatomical separation than shuffled controls (Fig. 5g), indicating that cell body position within a tectal t-type strongly influences its functional phenotype. Of note, t/f-clusters of the same f-types that were separated by t-type also showed significantly lower spatial overlap within some individual f-types and across f-types.

Finally, we explored whether the location of the cell body of an OT neuron was more informative of t-type identity than its functional properties. We performed support vector machine (SVM) classification on t-type on the basis of the nine marker genes, given either the anatomical cell body position or position in the eight-dimensional functional space as input. In line with the dependency of functional identity on anatomical localization, we found that the position of the cell body was a better statistical predictor for t-type identity than functional profile, and combining both features did not significantly increase predictive performance (Fig. 5h,i).

## Transcriptome and position govern morphology

**Fig. 6:**
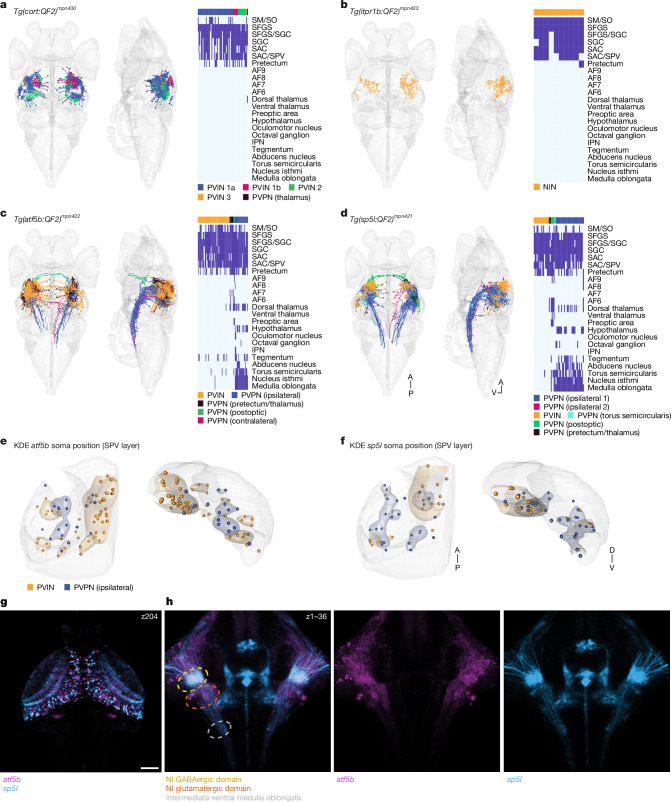
Neurons of a specific t-type may assume distinct morphologies and projection patterns. **a**, Sparsely labelled *cort* neurons were registered into the standard brain (*n* = 62). Right, anatomical matrix. AF, arborization field; IPN, interpeduncular nucleus; SAC, stratum album centrale; SGC, stratum griseum centrale; SM, stratum marginale; SO, stratum opticum. **b**, Sparsely labelled *itpr1b* neurons were registered into the standard brain (*n* = 10). Right, anatomical matrix. NIN, neuropil interneurons. **c**, Sparsely labelled *atf5b* neurons were registered into the standard brain (*n* = 77). Neurons are colour coded according to their m-type. Right, anatomical matrix. **d**, Sparsely labelled *sp5l*^*+*^ neurons were registered into the standard brain (*n* = 53). Right, anatomical matrix. A, anterior; P, posterior; V, ventral. **e**, *atf5b* interneurons (*n* = 49) and ipsilateral neurons (*n* = 18) were mirrored to the left hemisphere, and a KDE was measured according to their soma position, revealing separation of m-types along the anterior–posterior and dorsal–ventral axes. Transverse and coronal views of the SPV layer are shown. **f**, *sp5l* interneurons (*n* = 16) and ipsilateral neurons (*n* = 29) were mirrored to the left hemisphere, and a KDE was measured according to their soma position, revealing separation of m-types along the anterior–posterior and dorsal–ventral axes. **g**, Registered confocal stack of *atf5b* and *sp5l* transgenic fish. A single fish example of each transgenic line out of three specimens imaged. A single focal plane spanning the OT is shown. Scale bar, 50 μm. **h**, *z*-projection of the nucleus isthmi (NI) area, showing *atf5b* and *sp5l* projections forming collaterals at different parts of the nucleus isthmi.

**Extended Data Fig. 12:**
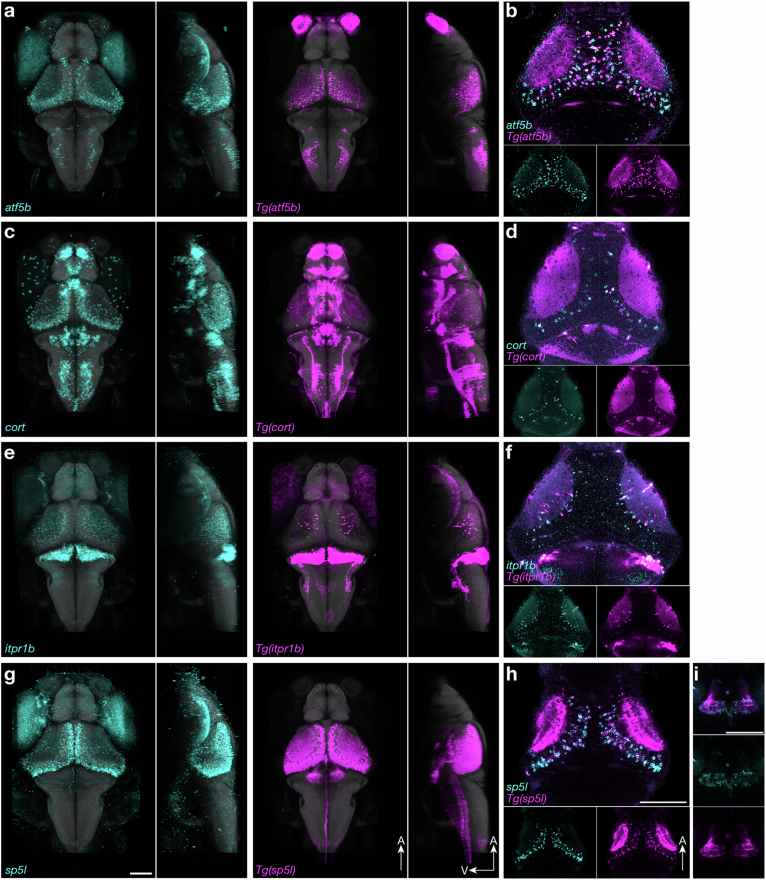
Comparison of transgenic expression with gene expression map. **a**, Average expression of *atf5b* labelled by in situ HCR (cyan) versus knock-in transgenic (Tg) labelling of *atf5b*+ neurons (magenta). The expression pattern of 3 individual fish was registered onto the *mapzebrain.org* atlas and averaged. **b**, Co-labelling of *atf5b* and *Tg(atf5b)*. A single tectum plane is shown. While not all the *atf5b* detected with HCR are labelled in the transgenic line, all the transgenic labelled neurons are HCR positive. This results from either variegation of the QF2/QUAS expression, or from neurons expressing the RNA but not the protein product. **c**, Average expression of *cort* labelled by in situ HCR (cyan) versus knock-in transgenic labelling of *cort*+ neurons (magenta). The expression pattern of 3 individual fish was registered onto the *mapzebrain.org* atlas and averaged. **d**, Co-labelling of *cort* and *Tg(cort)*. A single tectum plane is shown. **e**, Average expression of *itpr1b* labelled by in situ HCR (cyan) versus knock-in transgenic labelling of *itpr1b*+ neurons (magenta). The expression pattern of 3 individual fish was registered onto the *mapzebrain.org* atlas and averaged. **f**, Co-labelling of *itpr1b* and *Tg(itpr1b)*. A single tectum plane is shown. **g**, Average expression of *sp5l* labelled by in situ HCR (cyan) versus knock-in transgenic labelling of *sp5l*+ neurons (magenta). The expression pattern of 3 individual fish was registered onto the *mapzebrain.org* atlas and averaged. A weak expression of *sp5l* is detected in the caudal hypothalamus in comparison to the Tg line (see k). **h**, Co-labelling of *sp5l* and *Tg(sp5l)*. A single tectum plane is shown. **i**, Co-labelling of *sp5l* and *Tg(sp5l)*. A single plane of the caudal hypothalamus is shown.

**Extended Data Fig. 13:**
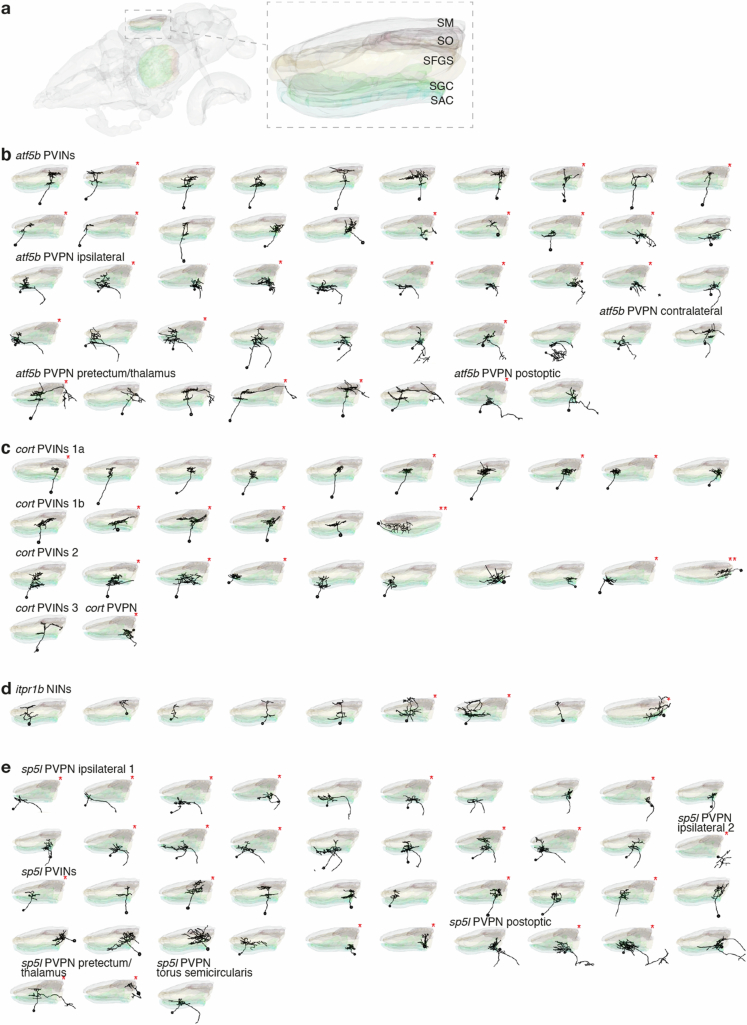
Variation of neuropil stratification patterns within t-types and m-types. **a**, Illustration of the larval zebrafish brain, highlighting the tectal neuropil layers. Abbreviations: SAC; stratum album centrale, SFGS; stratum griseum et fibrosum superficiale, SGC; stratum griseum centrale, SM; stratum marginale, SO; stratum opticum. **b**–**e**, Stratification patterns within the tectal neuropil of selected m-types, for each of the t-types examined. Axonal patterns of the projection neurons can be visualized in Fig. 6a–d and using mapzebrain.org brain atlas. *Right hemisphere located neurons, whose images were mirrored for visualization simplicity. **Lateral and not medial viewing angle.

Tectal neurons exhibit a rich diversity of morphologies, including specific dendritic and axonal targets^5,6,36–39^. To explore whether t-types are related to the different morphological types, we sparsely labelled single neurons in transgenic reporter lines, labelling the *atf5b*, *cort*, *itpr1b* and *sp5l* t-types (Extended Data Fig. 12), and traced their morphologies (Methods). The vast majority of *cort* neurons were inhibitory periventricular interneurons (PVINs), with cell bodies located in the SPV layer. The *cort*-expressing interneurons varied in their stratification patterns: two types were monostratified within the stratum griseum et fibrosum superficiale (SFGS) layer, showing narrow (PVIN 1a) or wide stratification patterns (PVIN 1b), whereas a third type bistratified in the SGC/SAC and SFGS layers (PVIN 2). We also observed one *cort* neuron projecting towards the dorsal thalamus (Fig. 6a and Extended Data Fig. 13c). The *itpr1b* neurons reside exclusively in the neuropil (Fig. 6b). Their neurites are often tristratified, reminiscent of the tectal pyramidal/type I neurons in adult cyprinid fish^36,39^, but additional morphologies were also observed in this transgenic line, including monostratified and bistratified neurons (Extended Data Fig. 13d).

The excitatory t-types *atf5b* and *sp5l* included specific subsets of ipsilaterally or contralaterally projecting neurons and interneurons with a range of dendritic stratification patterns (Fig. 6c,d and Extended Data Fig. 13b,e). Generally, we observed that the number of distinct projection and stratification patterns found for single neurons within a given transgenic line far exceeded the number of transcriptomic clusters expected for this marker. This rule applies to both the single-cluster marker genes investigated (*cort*, *itpr1b*) and the marker genes expressed in multiple clusters (*atf5b*, *sp5l*).

We asked whether we could further differentiate neurons with comparable projection patterns according to t-type. Registration of confocal images of *atf5b* and *sp5l* neurons highlighted the differences between their target domains (Fig. 6g,h): *sp5l* neurons have ipsilateral axon collaterals within the GABAergic domain of a tegmental nucleus, the nucleus isthmi^40^, whereas *atf5b* neurons form ipsilateral collaterals in its glutamatergic domain (Fig. 6h). As is the case for t/f-clusters, the morphologies of neurons belonging to the same t-type vary across the OT: both *atf5b* and *sp5l* cells tend to develop into interneurons in the anterior OT and into projection neurons in the posterior OT (Fig. 6e,f). In sum, this suggests that individual t-types accommodate a distinct range of morphologies and connectivities, arranged in a position-dependent manner across the anterior–posterior axis of the OT.

## Discussion

The shape, synaptic connectivity and function of a neuron—that is, its phenotype—are dictated, or at least bounded, by the genes it expresses. High-throughput barcoding and sequencing efforts promise to offer deep insights into brain function, provided that transcriptomic datasets can be read out at scale and eventually translated into wiring diagrams^41^. This formidable task is aided by the observation that single-cell transcriptomes can be clustered by similarity of their gene expression profiles, with clusters corresponding to individual cell types. Such dimensionality reduction allows researchers to focus on characterizing the connectivity and functional properties of reproducible sets of neurons, ‘cell type by cell type’ and across animals. Single-cell RNA sequencing and spatial transcriptomics have successfully matched cell types to specific brain regions^42–44^, developmental trajectories^45–49^ and specific functions^2,4,7,50^, although the functional assignment is less stringent and consistent^16,17,51^. However, there are at least two biological factors that confound this straightforward cell-type concept: position in the tissue and development.

First, transcriptionally similar neurons may differ locally in phenotype in brain areas that are regionally specialized, such as topographic maps, which are prevalent in the visual, auditory and somatosensory systems. For the retinotopically mapped OT, we find that transcriptomic cell types have overlapping sets of visual responses and morphologies, and these are organized along the spatial coordinates of the OT. We speculate that these regional differences in f-type distribution reflect their input and output heterogeneities. Such heterogeneities are well known and ethologically relevant because visual processing is adapted to stimulus statistics that are not uniform across the visual field^5,52,53^.

Second, unlike transistors on a microchip, neurons are not manufactured in a single sweep; they grow in number by cell division and connect by extending neurites, often over long distances, and with their growth cones searching for synaptic partners with matching molecules on their surface. Later-born neurons of the OT, which reside closer to the posterior marginal zone, are more likely to receive retinotopic information from the nasal retina. These neurons have a different set of possible synaptic partners than similar t-types that are born earlier and reside at the anterior OT, ultimately resulting in an asymmetric circuit layout. Additionally, synaptic partners are defined by expression of cell-surface and axon guidance molecules, which are transiently expressed during development^49,54^, increasing the phenotypic diversity within transcriptomically indistinguishable neurons born later.

Although our data only represent a single developmental time point, the spatial position in the OT can serve as a proxy for the neuron’s time of birth. Along the superficial-to-deep axis of the tectum, the transcriptomic layering that we identified correlates strongly with time of birth: early-born neurons come to reside closer to the surface of the SPV, as they are displaced by later-born neurons, which stay near the deep ventricular zone^21,22^. A similar temporally staggered development of cell types has been reported in vertebrate and invertebrate organs and tissues^29,55^. Thus, neurons are under the influence of dynamically changing local cues depending on when and where in the tissue they are born, differentiate and form connections. These dynamic cues may result from transient intrinsic gene expression among the similarly sequenced t-types born at different times, from local or temporal extrinsic signals, or from a combination of these factors.

An influence of birthdate and positional information on the expression of cell-type traits supports the notion that a limited number of global transcriptomic states are re-used and locally adapted to participate in specialized circuits and display divergent functional response profiles^56^. In this revised concept, cell types correspond to t-types and require further stratification based on position and development in order to be predictive for function and morphology. Although the repertoire of genetically distinguishable t-types is already enormous^7,12,13,57^, spatially and temporarily restricted cues can generate even more phenotypic variants of genetically programmed circuitry.

## Methods

### Zebrafish husbandry and maintenance

Zebrafish were kept at 28 °C under a day/night cycle of 14/10 h, pH of 7–7.5, and a conductivity of 600 µS. The following transgenic lines were used in this study: Wild-type fish of the TL strain, *Tg(elavl3:H2b-GCaMP6s)jf5*, *Tg(atf5b:QF2)mpn422*, *Tg(cort:QF2)mpn430*, *Tg(itpr1b:QF2)mpn423*, *Tg(sp5l:QF2)mpn421* and *Tg(QUAS:GCaMP6s)mpn164*. All larvae were produced by natural matings and raised until 6 or 7 days post fertilization (dpf) at 28 °C in Danieau’s solution in petri dishes. The animal experiments were performed under the regulations of the Max Planck Society and the regional government of Upper Bavaria (Regierung von Oberbayern), approved protocols: ROB-55.2-2532.Vet_03-19-86, ROB-55.2-2532.Vet_02-19-16, ROB-55.2-2532.Vet_02-21-93.

### Single-cell RNA sequencing

#### Tissue dissection

The 6 or 7 dpf wild-type larvae were anaesthetized in tricaine (one larva at a time), followed by removal of the eye and the skin around the brain using a fine Tungsten needle. Both OT hemispheres, together with the torus longitudinalis, were carefully dissected under a stereoscope and transferred into a 1.5 ml low-binding-protein tube containing ~50 µl PBS, and placed on ice. Cell dissociation was performed immediately after the dissection. Eleven dissection sessions were performed on different days, with 20–25 OT and torus longitudinalis dissected for each batch.

#### Cell dissociation

Cell dissociation was performed using papain (Papain Dissociation System, Worthington Biochemical Corporation). Five hundred microlitres of oxygenated papain was added to the 1.5 ml tube containing the 50 μl PBS and the dissected tissue, and incubated at 37 °C with superficial oxygen flow. The samples were gently pipetted every 10 min, and were fully dissociated after 45 min (a 2 μl sample was visually inspected to assess cell separation). The cells were then centrifuged for 10 min at 400*g*, at 4 °C, using a swinging bucket centrifuge. The supernatants were removed, and the cells were resuspended in 260 μl of equilibrated ovomucoid solution and 30 μl of DNaseI. The cells were then centrifuged for 10 min at 400*g*, at 4 °C, the supernatants were removed, and the cells were resuspended in 500 μl PBS and 0.04% BSA. This step was repeated once again, after which the cells were filtered using 30 μm cell strainer. Finally, the cells were centrifuged, and the supernatants were removed, leaving 80 μl in which the cells were pipette gently. Five microlitres of the dissociated cells were transferred into a new 1.5 ml low-binding protein tube and mixed with 13 μl PBS, 0.04% BSA and 0.4% Trypan blue for cell counting and viability assessment.

#### Library preparation and sequencing

The dissociated cells were loaded onto a commercially available droplet-based single-cell barcoding system (10x Chromium Controller, 10x Genomics). The Chromium Single Cell 3′ Reagent Kit v3 (10x Genomics) was used to prepare single-cell 3′ barcoded cDNA and Illumina-ready sequencing libraries according to the manufacturer’s instructions. The cDNA libraries were sequenced using an Illumina HiSeq 2500 machine (batches 1–8) or Illumina NovaSeq 6000 (batches 9–11), with a mean of ~76,000 reads per cell over the 11 batches.

#### Quality check, batch correction and clustering analysis

The sequenced data were processed using CellRanger-7.1.0 (filtering, barcode and unique molecular identifier (UMI) counting) with default command line options. The sequenced reads were aligned to the zebrafish GRCz11 genome assembly (Ensembl release 98). To prevent confounding effects of the tissue dissection and cell dissociation on gene expression analysis, immediate early genes (IEGs) that were induced during the procedure^60^ were removed prior to the analysis. We have curated a list of 43 zebrafish IEGs (Supplementary Table 2), and excluded them from the cell–gene matrix. The data from each of the 11 batches were filtered using the Seurat R package version 5.1.0^61^, to ensure analysis of high quality cells, including filtering out cells expressing less than 600 or higher than 4,000 genes, cells with more than 7,500 UMIs, or containing higher than 10% mitochondrial genes. Additionally, to eliminate contamination from adjacent tissues, cells expressing the habenula marker genes *gng8* and *kiss1*, as well as cells expressing the lens crystallin gene *crygm2d13* above 0.5, were excluded. The data were then normalized using the Seurat LogNormalize method with a scale factor of 10,000. A set of 2,000 variable features were identified using the vst selection method, and the data were scaled using the ScaleData command. The detected variable features were used for principal component analysis. In order to filter out doublet cells from the analysis, initial clustering was performed on each batch separately, and the clustering information was used as an input for DoubletFinder^62^. Finally, the 11 batches were merged into a single Seurat object, and cells identified as doublets by DoubletFinder (*n* = 5,931) were filtered out. The resulting dataset contained 45,766 cells with a mean of 1,224 genes per cell and 2,420 UMIs. We then performed batch correction using Harmony^63^, grouping the variables according to their original batch followed by dimensionality reduction with UMAP^64^. Clustering analysis was performed by applying the Seurat FindNeighbors command with reduction=”harmony” and dims = 25 and the FindCluster command with resolution set to 0.6 (sub-clustering resolution was: neurons = 1, excitatory neurons = 1.2, inhibitory neurons = 1.2). The clustering resolutions were defined using clustering trees generated with the R package Clustree, together with SC3 stability scores^65,66^. Following the separation of excitatory and inhibitory cells, and prior to their respective clustering, cells expressing *gad1b* above 0.8 were filtered out from the excitatory dataset, while cells expressing either *slc17a6a* (also known as *vglut2b*) or *slc17a6b* (also known as *vglut2a*) above 0.8 were filtered out from the inhibitory dataset. After manual inspection of marker gene expression, clusters e24, e28 and e31 were split from a single cluster, as well as clusters i3 and i16, using the FindSubCluster command with resolutions of 0.3 and 0.25, respectively. To evaluate clustering correlation, genes were ranked according to their specificity score, calculated using the genesorteR R package^67^, and correlation matrices were generated according to the top ten scored genes per cluster. The vast majority of clusters showed low correlation with other clusters, representing unique t-types. Cluster robustness was evaluated by measuring pairwise Jaccard index of cells grouped together under different clustering parameters using the R package scclusteval^68^. Additionally, cluster stability was evaluated by bootstrapping as described in Tang et al. 2021^68^. Eighty per cent of the cells were sampled and reclustered. The highest Jaccard index was recorded for matching clusters for each subsample. This procedure was repeated 100 times and plotted as a Jaccard Raincloud plot. In most instances, we observed that clusters with small numbers of cells were scored low, as a result of infrequent sampling of those cells.

Generally, we took a conservative approach when defining and including clusters, which were then followed by validation of marker genes in situ.

Finally, differentially expressed genes were identified using the Wilcoxon Rank Sum test integrated in the Seurat FindAllMarkers command. Pseudotime analysis was performed using Monocle3^69^.

### Multiplexed and iterative in situ hybridization chain reaction

HCR experiments were performed on *Tg(elavl3:H2b-GCaMP6s)jf5* (to enable registration onto a common coordinate system) or the transgenic knock-in larvae labelling *atf5b*, *cort*, *itpr1b* and *sp5l* expressing cells. All HCR reagents including probes, hairpins and buffers were purchased from Molecular Instruments. The staining was performed according to previously published and modified protocol^28,70^. The larvae were anaesthetized in 1.5 mM tricaine and fixed with ice-cold 4% paraformaldehyde/Dulbecco’s PBS (DPBS) overnight at 4 °C with gentle shaking. The following day, larvae were washed 3 times for 5 min with DPBST (1× Dulbecco’s PBS + 0.1% Tween-20) to stop fixation, followed by a short 10-min treatment with ice-cold 100% methanol at −20 °C to dehydrate and permeabilize the tissue samples. Next, rehydration was performed by serial washing of 50% methanol/50% DPBST and 25% methanol/75% DPBST for 5 min each and finally 5× 5 min in DPBST. Ten to twelve larvae were transferred into a 1.5 ml tube and pre-hybridized with pre-warmed hybridization buffer for 30 min at 37 °C. Probe solution was prepared by transferring 2 pmol of each HCR probe set (2 µl of 1 µM stock) to 500 µl of hybridization buffer at 37 °C. The hybridization buffer was replaced with probe solution, and the samples were incubated for 12–16 h at 37 °C with gentle shaking. To remove excess probes, larvae were washed 4× 15 min with 500 µl pre-warmed probe wash buffer at 37 °C. Subsequently, larvae were washed 2× 5 min with 5× SSCT (5× sodium chloride sodium citrate + 0.1% Tween-20) buffer at room temperature. Next, pre-amplification was performed by incubating the samples in 500 µl of amplification buffer for 30 min at room temperature. Separately, 30 pmol of hairpin h1 and 30 pmol of hairpin h2 were prepared by snap-cooling 10 µl of 3 µM stock by incubating the hairpins in 95 °C for 90 s, and cooling down to room temperature in a dark environment. After cooling down for 30 min, hairpin solution was prepared by transferring the h1 and h2 hairpins to a 500 µl amplification buffer. The pre-amplification buffer was removed and the samples were incubated in the hairpin solution for 12–16 h in the dark at room temperature. Excess hairpins were washed the next day 3× 20 min using 5× SSCT at room temperature. Larvae were then stored long-term at 4 °C in 5× SSCT until imaging.

For iterative HCR staining, the HCR probes and hairpins were stripped using DNAseI treatment. The imaged fish were placed separately in 1.5 ml tubes and incubated in a mix of 5 µl 10x reaction buffer (Invitrogen AM2238), 5 µl Turbo DNAseI (final concentration 0.2 U µl^−1^, Invitrogen AM2238) and 40 µl DPBS for 4 h at 37 °C. The samples were then washed 3 × 5 min with DPBST and the complete removal of the HCR signal was validated under a confocal microscope. The HCR stripped fish were kept separated in 1.5 ml tubes and underwent another round of HCR staining and imaging, beginning from the pre-hybridization step.

HCR data were registered onto the standard brain as previously described^28^ using Advanced Normalization Tools (ANTs)^71^. For the functional HCR experiments, where iterative HCR stainings were performed, each iteration was registered to the first iteration of the same animal. A total of 3 HCR rounds were performed on single animals without any noticeable morphological distortions or reduction of the endogenous fluorescence of the *Tg(elavl3:H2b-GCaMP6s)* used as the reference registration channel.

### HCR image registration

All HCR and immunostaining images were aligned onto the mapzebrain.org *Tg(elavl3:H2b-GCaMP6s)* standard brain^27,28^.

The ANTs registration command used was:

antsRegistration -d 3 --float 1 -o [${output1},${output2}] -- interpolation WelchWindowedSinc --use-histogram-matching 0 -r [${template},${input1},1] -t rigid[0.1] -m MI[${template},${input1},1,32,Regular,0.25] -c [200×200×200×0,1e-8,10] -- shrink-factors 12×8×4×2 --smoothing-sigmas 4×3×2×1vox -t Affine[0.1] -m MI[${template},${input1},1,32,Regular,0.25] -c [200×200×200×0,1e-8,10] -- shrink-factors 12×8×4×2 --smoothing-sigmas 4×3×2×1 -t SyN[0.01,6,0.0] -m CC[${template},${input1},1,2] -c [200×200×200×200×10,1e-7,10] --shrink- factors 12×8×4×2×1 --smoothing-sigmas 4×3×2×1×0 followed by applying the transformation files on the HCR image channels using the ANTs command:

antsApplyTransforms -d 3 -v 0 -- float -n WelchWindowedSinc -i ${input3} -r ${template} -o ${output4} -t ${output1}1Warp.nii.gz -t ${output1}0GenericAffine.mat

All the registered HCR data used in this study are publicly available through the *mapzebrain.org* atlas.

### Confocal imaging

HCR-labelled samples were embedded in 2% low-melting agarose in 1× DPBS (Dulbecco’s PBS) and imaged using an upright Zeiss LSM700 confocal scanning microscope with a 20× water immersion objective. The images were acquired with Zeiss ZEN 2012 SP1 software (Release Version 8.1.6.484). *z*-stacks, comprising 2 tiles (or 1 tile for functional HCR experiment), were taken and stitched to produce a final image with size of 1,039 × 1,931 pixel (463.97 × 862.29 µm, 1 µm in *z*).

### Pixel intensity quantification of HCR data

For each HCR-labelled gene, three larvae were imaged and registered as described above. A maximum intensity projection was generated for each image for planes z289–z291. Coronal sections were generated using the Fiji ‘reslice’ option^72^, and the maximum intensity projection was generated for planes x463–x465. ROIs spanning the SPV for both the transverse and coronal section were used to measure the pixel intensity profile using a custom Fiji macro. The data were analysed and plotted using R.

### Tissue localization of cell bodies with HCR signal

Cell centroids in the HCR data were manually labelled using napari points layer tool^73^, by examination of HCR signal together with the nuclear labelling of the *Tg(elavl3:H2b-GCaMP6s)*. For the functional imaged-HCR-labelled animals, the experimenter was blinded to the functional trace of the labelled centroids. For identifying co-labelled submarkers in *atf5b*- and *sp5l*-expressing cells, polygons were first manually drawn using napari around the cells expressing the main markers. The expression of submarkers were manually determined by labelling positive centroids within the main polygons. The centroids were then registered onto the standard brain using ANTs and the R package ANTsR. To define the molecular layers of the OT, the NNDs between centroids were measured in 3D using the R package spatstat^74^. The centroids were visualized using the R package Plotly, hierarchically clustered using complete linkage method implemented in the hclust function with default parameters, and plotted using the R package pheatmap^75^.

### Functional imaging of *Tg(elavl3:H2b-GCaMP6s)*

Two-photon functional calcium imaging was performed on 6–8 dpf *Tg(elavl3:H2b-GCaMP6s)* larvae that expresses a nuclear calcium indicator in all neurons, without paralysis or anaesthesia. The animals were embedded in 2% agarose and mounted onto the stage of a modified two-photon moveable objective microscope (MOM, Sutter Instrument, with resonant-galvo scanhead) with a 20× objective (Olympus XLUMPLFLN, NA 1.0). The data were acquired with ScanImage 5.6 software. Fish that drifted along the dorsoventral axis in the preparation were excluded from analysis. Volumetric imaging of the tectum was performed with a custom-built remote focusing arm^34^. Refocusing through the remote arm enabled rapid sequential imaging of 6 planes (512 × 512 pixels) spanning 60–100 µm of the tectum along the dorsoventral axis at 5 volumes per second. In each fish neuronal activity in the tectum was recorded over 2× 10-min sessions to cover the whole tectum, resulting in 12 imaging planes per fish in total. Laser power out of the objective ranged from 10 mW to 15 mW.

### Functional imaging of cell-type-specific transgenic lines

Animals were embedded and placed under a commercial two-photon laser scanning microscope (Femtonics) or custom-built two-photon microscope as described above^34,76^. Sequential single-plane imaging at either 5 fps (custom-built microscope) or between 1–1.5 fps (Femtonics) was performed at 4–10 different depths along the dorsoventral axis of the specimen for a 10-min session each, with the same stimulus set as detailed below. No anatomical stack for registration was acquired from these animals.

### Visual stimulation

Visual stimuli were designed with PsychoPy and projected by an LED projector (Texas Instruments, DLP Lightcrafter 4500, with 561 nm long-pass filter) from below onto Rosco Tough Rolux 3000 diffusive film water-immersed in a 10-cm petri dish. The embedded animal was placed into a 6 cm petri dish on top of the diffusive paper in the larger dish, with a spacer in between that ensured a water film between the diffusive paper and the smaller petri dish. The fish was placed at 12 mm distance from the projection screen. The fish head was manually centred in the imaging chamber, aided by projecting crosshairs on the screen. Stimuli were shown in a fixed sequence: gratings, dot motion (8×, see below for details), gratings, OFF ramp, ON ramp, looming (2×). All stimuli consisted of black features on a red background to not interfere with the green GCaMP6s fluorescence signal. Individual stimulus presentations were separated by 20 s inter-trial intervals, except for the two loom stimuli, for which a 1-min interval was used. The whole duration of the stimulus protocol was 10 min.

#### Moving gratings

Gratings moving caudo-rostrally with respect to the fish were shown once at the beginning and after the dot stimuli with 20 mm width and 2 Hz temporal frequency for 20 s.

#### Dot motion

A black dot moving on a circular trajectory (radius, 18 mm) was shown starting in front of the fish. The dot stimulus moved either in discrete jumps at 1.5 Hz or in a perceptually smooth displacement at 60.0 Hz (projector frame rate) with the same overall speed of 5 mm s^−1^ (15.9° s^−1^), resulting in a stimulus duration of 22.6 s. Each frequency was presented using a dot diameter of 4 mm (12.7°). Both clockwise and counter-clockwise presentations were shown, resulting in four different stimuli. The whole dot stimulus set was repeated twice in each session, so eight dot stimuli were shown in succession.

#### OFF/ON ramp

Whole-field luminance of the projected blank image was decreased to zero over the course of 2 s, and ramped up to normal background luminance within 2 s after 20 s delay.

#### Looming

A linearly disk was displayed with expansion from 0.6° to 110° in 83 ms centred below the fish.

### *z*-stack acquisition, registration and ROI matching

After each functional recording, a high-resolution 2-photon *z*-stack of a large subvolume of the brain, including the full midbrain region, was taken (1,024 × 1,024 pixels, 1 µm in *z*, 835 nm laser wavelength, plane averaging 100×). Each time series average of the 12 imaging planes were registered to this stack using the scikit-learn template matching algorithm. The 2-photon brain volume was then registered to the volume of the first round HCR confocal imaging as described in ‘HCR image registration’. To transform functional ROIs from 2-photon space into HCR space, ROI pixel coordinates were transformed first from imaging plane reference frame to 2-photon *z*-stack reference frame and finally to the first round HCR reference frame the by running the ANTs command antsApplyTransformsToPoints twice using the respective transformation matrices from each registration step. HCR cell centroid annotations from each round were transformed into the first round HCR reference frame, and all coordinates were used as seeds for generating 3 × 3 pixel volumes that were overlaid with registered functional ROI pixels. HCR centroids were assigned to a functional ROI based on the largest fractional overlap. Finally, all assigned as well as unassigned functional ROIs were transformed to the standard brain as described above.

### Analysis of two-photon imaging data

Suite2p^77^ was used for motion correction, ROI detection, ROI classification, and signal extraction (time constant tau = 7 s, diameter = 4 pixels). In detail, raw volume recording files were deinterleaved into individual time series for each imaging plane. Rigid and non-rigid motion correction was performed with suite2p on a low-pass filtered time series in *xy* (gaussian, sigma = 4). The motion-correction shifts were applied to the raw imaging time series. ROIs were detected on fivefold downsampled and motion-corrected time series and fluorescence traces were extracted using the average pixel intensities of ROIs over time. All functional ROIs were initially thresholded based on the built-in suite2p classification algorithm iscell and an anatomical tectal mask drawn in the standard brain (mapzebrain.org).

#### Response score

For each stimulus, a regressor was constructed by convolving a boxcar function with an exponential decay kernel that mimics the H2B::GCaMP6s off-kinetics. The resulting 8 regressors were separately fitted to the calcium trace of each functional ROI, using a linear regression model on the stimulus time window. The response score was calculated as the product of the regression coefficient (equivalent to d*F*/*F*) and the coefficient of determination *R*^2^. For clustering and dimensionality reduction (Fig. 5), the analysis included all functional ROIs that scored above the 95th percentile of the population response score for any of the visual stimuli. Response score vectors of all high-scoring neurons excluding any t-type positive ROIs (n = 7094) were scaled to unit variance and zero mean of the overall tectal population for subsequent analysis.

#### Hierarchical clustering and embedding

Functional clusters were identified in a two-step analysis: First, exemplars of the 7,094 high-scoring ROIs in 8-dimensional functional space were identified using affinity propagation (sklearn.cluster.AffinityPropagation, default parameters). Exemplars with less than 10 associated ROIs were excluded from subsequent analysis. The remaining 169 exemplars were clustered using hierarchical clustering (scipy.cluster.hierarchy.linkage, method=‘complete’, metric=‘correlation’; scipy.cluster.hierarchy.fcluster, criterion=‘maxclust’). In parallel, the 2-dimensional embedding for visualizing functional space was computed from the 8-dimensional response vectors of all high-scoring ROIs using UMAP (n_neighbors=25, min_dist=0.7, metric=‘euclidean’). Functional ROIs matched with HCR labels or functional ROIs from transgenic lines were transformed into the initial UMAP space by applying the UMAP transform function to their respective response vectors.

#### Anatomical overlap metric

3D coordinates of each t/f-subcluster that contained more than 10 ROIs were used to generate a gaussian kernel density estimate (KDE) with scipy.stats.gaussian_kde (bandwidth = 0.75). For computing pairwise overlap, the KDEs were sampled, normalized, and the minimum of the joint KDEs was taken at each point. The overlap metric is bounded from 0 to 1 (0 if no overlap, near 1 if the same). Controls were generated by shuffling t-type labels of all ROIs in the whole dataset.

#### t-type classification

Response scores of t-type+ ROIs were scaled to unit variance and zero mean. An SVM classifier (sklearn.svm.SVC, kernel=‘rbf’, gamma=‘scale’, C=1) was trained on 90% of all t-type+ response vectors and gene labels. To counter the uneven distribution of t-types, the training data were upsampled to 1,000 samples per t-type. Evaluation of the classifier performance was done on 10% holdout test data. This process was repeated 20 times with permutated training/test data splits. The same classification was performed with anatomical centroid positions of t-type+ ROIs as dependent variables as well as using both response vectors and anatomical positions. Each classification was run again with shuffled marker gene labels as a negative control.

### Generation of knock-in transgenic lines

The knock-in lines *Tg(sp5l:QF2)mpn421*, *Tg(atf5b:QF2)mpn423*, *Tg(itpr1b:QF2)mpn424* and *Tg(cort:QF2)mpn430* were generated by locus-specific insertions using CRISPR–Cas9 and the GeneWeld approach^78^. Guide RNA (gRNA) target sequences were identified using the CCTop tool^79^. The gRNA target sequences are: *atf5b*, 5′-ATTTGGACGTCATGCTCCAGAGG-3′; *cort*, 5′-GCCCCTGGAGTCCCGTCTGG-3′; *itpr1b*, 5′-CATCTGCTCCCTGTATGCGGAGG-3′; *sp5l*, 5′-AGGCTCGCAGCTCCCTTACGAGG-3′. Short homology sequences of 48 bp spanning the upstream and downstream of gRNA site were ordered as complementary oligonucleotides (MWG) and cloned into donor plasmids using the GoldenGATEway strategy^80^. Universal gRNAs (ugRNA)^78^ were introduced into the donor to release the insert from plasmid after injection. The order of components of all the donor constructs was the following: ugRNA, upstream homology arm, short GSG linker, T2A, QF2, polyA signal, downstream homology arm and second ugRNA (inverted). CRISPR–Cas9 ribonucleoprotein complex was prepared at a concentration of 1.5 μM as described before^4^. The gRNA was produced by annealing customized CRISPR RNA (crRNA) (IDT, Alt-R CRISPR–Cas9 crRNA) with *trans-*activating crRNA (tracrRNA) (IDT, 1072533) in annealing buffer (IDT, 11-05-01-12). The gRNA was incubated with Cas9 protein (IDT, 1081060) for 15 min at 37 °C and the donor plasmid was added to the injection mix, at a final concentration of 20 ng μl^−1^. The CRISPR–Cas9 mix was injected into *Tg(QUAS:epNTR-RFP)mpn165* embryos at the single-cell stage. Positive transient expressor fish were raised and screened at adulthood for germline transmission.

### Cellular tracing and morphology analysis

Single neurons were sparsely labelled either during the knock-in generation procedure in mosaic F_0_ animals, or by transiently microinjecting 12.5 ng μl^−1^ QUAS:eGFP-caax plasmid into the *Tg(atf5b:QF2)mpn423*, *Tg(cort:QF2)mpn430*, *Tg(itpr1b:QF2)mpn424* or *Tg(sp5l:QF2)mpn421* transgenic embryos at the single-cell stage. At 6 dpf the injected larvae were anaesthetized in a lethal dose of tricaine, fixed in 4% paraformaldehyde and immunostained with mouse anti-ERK1/2 (Cell Signaling Technology 4696S, 1:250 dilution) and Chicken anti-GFP (Invitrogen A10262, 1:250 dilution), according to a previously published protocol^27^.

Confocal imaging was performed as described above. Individual neurons were semi-automatically traced using the freeware NeuTube^81^, saved as SWC files and registered to the mapzebrain.org atlas using ANTs as described above and previously^27^.

The traced neurons were plotted using the R package natverse^82^ and manually clustered according to the projection terminals or neuropil stratification patterns. A matrix for the anatomical crossing areas for each neuron was generated with the standard brain atlas (mapzebrain.org), and the heat maps were plotted using the R package pheatmap^75^. Soma positions were used to generate a gaussian kernel density estimate using the R packages misc3d and oce^83^ and plotted using the R package rgl^84^.

### Reporting summary

Further information on research design is available in the Nature Portfolio Reporting Summary linked to this article.

## Online content

Any methods, additional references, Nature Portfolio reporting summaries, source data, extended data, supplementary information, acknowledgements, peer review information; details of author contributions and competing interests; and statements of data and code availability are available at 10.1038/s41586-024-08518-2.

## Supplementary information

Reporting Summary

Supplementary Table 1 Top differentially expressed genes per cluster.

Supplementary Table 2 List of IEGs excluded from the analysis.

## Data Availability

Single-cell RNA sequencing raw and processed data files are available through the NCBI Gene Expression Omnibus (GEO) under the accession number GSE269232. The two-photon calcium imaging data are accessible from 10.5281/zenodo.14146655 (ref. ^85^). All HCR registered images and neuronal tracings are available at https://mapzebrain.org.

## Code availability

All the Python, R and ImageJ scripts used in this study are accessible at 10.5281/zenodo.14146655 (ref. ^85^).
