# Supplementary material for: Transcriptomic neuron types vary topographically in function and morphology
Source: Nature. 2025 Feb 12;638(8052):1023–33. doi: 10.1038/s41586-024-08518-2 (PMC11864986; doi:10.1038/s41586-024-08518-2)
Supplement: Supplementary file 1 — Reporting Summary [file 41586_2024_8518_MOESM1_ESM.pdf]

nature portfolio

Corresponding author(s): Herwig Baier
Last updated by author(s): Nov 12, 2024

# Reporting Summary

Nature Portfolio wishes to improve the reproducibility of the work that we publish. This form provides structure for consistency and transparency in reporting. For further information on Nature Portfolio policies, see our Editorial Policies and the Editorial Policy Checklist.

## Statistics

For all statistical analyses, confirm that the following items are present in the figure legend, table legend, main text, or Methods section.

| n/a | Confirmed | |
|---|---|---|
| ☐ | ☒ | The exact sample size ($n$) for each experimental group/condition, given as a discrete number and unit of measurement |
| ☐ | ☒ | A statement on whether measurements were taken from distinct samples or whether the same sample was measured repeatedly |
| ☐ | ☒ | The statistical test(s) used AND whether they are one- or two-sided<br>*Only common tests should be described solely by name; describe more complex techniques in the Methods section.* |
| ☐ | ☒ | A description of all covariates tested |
| ☐ | ☒ | A description of any assumptions or corrections, such as tests of normality and adjustment for multiple comparisons |
| ☐ | ☒ | A full description of the statistical parameters including central tendency (e.g. means) or other basic estimates (e.g. regression coefficient) AND variation (e.g. standard deviation) or associated estimates of uncertainty (e.g. confidence intervals) |
| ☐ | ☒ | For null hypothesis testing, the test statistic (e.g. $F$, $t$, $r$) with confidence intervals, effect sizes, degrees of freedom and $P$ value noted<br>*Give P values as exact values whenever suitable.* |
| ☒ | ☐ | For Bayesian analysis, information on the choice of priors and Markov chain Monte Carlo settings |
| ☒ | ☐ | For hierarchical and complex designs, identification of the appropriate level for tests and full reporting of outcomes |
| ☒ | ☐ | Estimates of effect sizes (e.g. Cohen's $d$, Pearson's $r$), indicating how they were calculated |

*Our web collection on statistics for biologists contains articles on many of the points above.*

## Software and code

Policy information about availability of computer code

| | |
|---|---|
| Data collection | Zeiss ZEN 2012 SP1 Release Version 8.1.6.484, ScanImage 5.6 |
| Data analysis | CellRanger-7.1.0<br>R version 4.4.1<br>R packages: Seurat 5.1.0, Harmony 1.2, DoubletFinder 2.0.4, harmony 1.2.0, Clustree 0.5.1, scclusteval 0.0.0.9, genesorteR 0.4.3, Monocle3 1.3.7, ANTsR 0.6.1, spatstat.sparse 3.1, pheatmap 1.0.12 plotly 4.10.4, natverse 0.2.4, oce 1.8-3, misc3d 0.9-1, rgl 1.3.1 and additional packages.<br>Python version 3.11<br>Python packages: Suite2p 0.14.4, scikit-image 0.22.0, scikit-learn 1.2.2, scipy 1.11.1, pandas 2.1.1, numpy 1.25.2, umap-learn 0.5.4, and additional packages.<br>The additional R and python packages are listed together with the deposited code.<br>ANTs 1.9<br>ImageJ 1.53c<br>NeuTube 1.0 |

For manuscripts utilizing custom algorithms or software that are central to the research but not yet described in published literature, software must be made available to editors and reviewers. We strongly encourage code deposition in a community repository (e.g. GitHub). See the Nature Portfolio guidelines for submitting code & software for further information.

## Data

Policy information about availability of data

All manuscripts must include a data availability statement. This statement should provide the following information, where applicable:

- Accession codes, unique identifiers, or web links for publicly available datasets
- A description of any restrictions on data availability
- For clinical datasets or third party data, please ensure that the statement adheres to our policy

Single-cell RNA sequencing raw and processed data files are available through NCBI's Gene Expression Omnibus (GEO) under the accession number GSE269232. Two-photon calcium imaging data will be made publicly available upon publication. All HCR registered images and neuronal tracings are available at mapzebrain.org.

## Research involving human participants, their data, or biological material

Policy information about studies with human participants or human data. See also policy information about sex, gender (identity/presentation), and sexual orientation and race, ethnicity and racism.

| | |
|---|---|
| Reporting on sex and gender | NA |
| Reporting on race, ethnicity, or other socially relevant groupings | NA |
| Population characteristics | NA |
| Recruitment | NA |
| Ethics oversight | NA |

Note that full information on the approval of the study protocol must also be provided in the manuscript.

# Field-specific reporting

Please select the one below that is the best fit for your research. If you are not sure, read the appropriate sections before making your selection.

☒ Life sciences ☐ Behavioural & social sciences ☐ Ecological, evolutionary & environmental sciences

For a reference copy of the document with all sections, see nature.com/documents/nr-reporting-summary-flat.pdf

# Life sciences study design

All studies must disclose on these points even when the disclosure is negative.

| | |
|---|---|
| Sample size | The sample size for each of the single-cell RNA sequencing batches was limited to the number of animals that could be dissected within a two-hour time window. This limitation was implemented to prevent the effects of cell stress on RNA detection. Eleven dissection batches were performed, resulting in 45,766 sequenced cells, which corresponds to >7x coverage of cells in a single tectal hemisphere. For functional imaging analysis, a sample size of six animals was chosen to ensure sufficient statistical power and to balance with practical considerations when combined with subsequent HCR analysis. Neuronal morphology tracings were performed on 10-50 animals, depending on the gene of interest, and its labeling efficiency. |
| Data exclusions | No individual data points were excluded from the analysis. Animals damaged during the embedding or imaging were not analyzed further as described in the methods section. |
| Replication | For the scRNA-seq, Eleven dissection batches were performed on different days, with 20-25 OTs and TLs dissected during each batch. For functional imaging analysis, 3 animals were recorded each day, over two different recording days. The micro injections for neuronal morphology tracings were performed for 2-4 times per genes of interest, on different egg clutches. All replication attempts were successful. |
| Randomization | Animals were randomly chosen from genetically similar clutches. |
| Blinding | For the scRNA-seq and functional imaging, the experimenters were not blinded to experimental conditions or genotype of the animals. The analysis of gene expression data and functional responses is done through computational methods, eliminating the possibility of subjective bias from researchers. For the manually labeled HCR positive neurons, the experimenter was blinded to the functional trace of the labeled centroids. |

# Reporting for specific materials, systems and methods

We require information from authors about some types of materials, experimental systems and methods used in many studies. Here, indicate whether each material, system or method listed is relevant to your study. If you are not sure if a list item applies to your research, read the appropriate section before selecting a response.

## Materials & experimental systems

| n/a | Involved in the study |
| --- | --- |
| ☐ | ☒ Antibodies |
| ☒ | ☐ Eukaryotic cell lines |
| ☒ | ☐ Palaeontology and archaeology |
| ☐ | ☒ Animals and other organisms |
| ☒ | ☐ Clinical data |
| ☒ | ☐ Dual use research of concern |
| ☒ | ☐ Plants |

## Methods

| n/a | Involved in the study |
| --- | --- |
| ☒ | ☐ ChIP-seq |
| ☒ | ☐ Flow cytometry |
| ☒ | ☐ MRI-based neuroimaging |

# Antibodies

| | |
| --- | --- |
| Antibodies used | GFP polyclonal antibody, Invitrogen, Catalog number: A10262, Lot number: 2480084. 1:250 dilution.<br>p44/42 MAPK (Erk1/2) (L34F12) mAb, Cell Signaling Technology, Catalog number: 4696S, Lot number: 0022. 1:250 dilution. |
| Validation | Previously validated for zebrafish use (see manufacturer's website and Kunst et al. 2019). |

# Animals and other research organisms

Policy information about studies involving animals; ARRIVE guidelines recommended for reporting animal research, and Sex and Gender in Research

| | |
| --- | --- |
| Laboratory animals | The zebrafish lines used were: wild-type fish of the TL strain, Tg(elavl3:H2b-GCaMP6s)jf5, Tg(atf5b:QF2)mpn422, Tg(cort:QF2)mpn430, Tg(itpr1b:QF2)mpn423, Tg(sp5l:QF2)mpn421, and Tg(QUAS:GCaMP6s)mpn164. Animal ages were either 6 or 7 days post-fertilization (as specified in the methods). |
| Wild animals | No wild animals were used in the study. |
| Reporting on sex | The sex of the animals cannot be determined at this early age. |
| Field-collected samples | No field collected samples were used in the study. |
| Ethics oversight | Max Planck Society and the regional government of Upper Bavaria (Regierung von Oberbayern) |

Note that full information on the approval of the study protocol must also be provided in the manuscript.

# Plants

| | |
| --- | --- |
| Seed stocks | NA |
| Novel plant genotypes | NA |
| Authentication | NA |
